# Up regulation in gene expression of chromatin remodelling factors in cervical intraepithelial neoplasia

**DOI:** 10.1186/1471-2164-9-64

**Published:** 2008-02-04

**Authors:** Ashleen Shadeo, Raj Chari, Kim M Lonergan, Andrea Pusic, Dianne Miller, Tom Ehlen, Dirk Van Niekerk, Jasenka Matisic, Rebecca Richards-Kortum, Michele Follen, Martial Guillaud, Wan L Lam, Calum MacAulay

**Affiliations:** 1 Cancer Genetics & Developmental Biology, British Columbia Cancer Research Centre, Vancouver, BC, Canada; 2 Pathology and Laboratory Medicine, The University of British Columbia, Vancouver, BC, Canada; 3 Obstetrics and Gynaecology, The University of British Columbia, Vancouver, BC, Canada; 4 Gynecologic Oncology, British Columbia Cancer Agency, Vancouver, BC, Canada; 5 Pathology, British Columbia Cancer Agency, Vancouver, BC, Canada; 6 Bioengineering, Rice University, Houston, Texas, USA; 7 Gynecologic Oncology, The University of Texas, M.D. Anderson Cancer Center, Houston, Texas, USA; 8 Cancer Imaging, British Columbia Cancer Research Centre, Vancouver, BC, Canada

## Abstract

**Background:**

The highest rates of cervical cancer are found in developing countries. Frontline monitoring has reduced these rates in developed countries and present day screening programs primarily identify precancerous lesions termed cervical intraepithelial neoplasias (CIN). CIN lesions described as mild dysplasia (CIN I) are likely to spontaneously regress while CIN III lesions (severe dysplasia) are likely to progress if untreated. Thoughtful consideration of gene expression changes paralleling the progressive pre invasive neoplastic development will yield insight into the key casual events involved in cervical cancer development.

**Results:**

In this study, we have identified gene expression changes across 16 cervical cases (CIN I, CIN II, CIN III and normal cervical epithelium) using the unbiased long serial analysis of gene expression (L-SAGE) method. The 16 L-SAGE libraries were sequenced to the level of 2,481,387 tags, creating the largest SAGE data collection for cervical tissue worldwide. We have identified 222 genes differentially expressed between normal cervical tissue and CIN III. Many of these genes influence biological functions characteristic of cancer, such as cell death, cell growth/proliferation and cellular movement. Evaluation of these genes through network interactions identified multiple candidates that influence regulation of cellular transcription through chromatin remodelling (*SMARCC1*, *NCOR1*, *MRFAP1 *and *MORF4L2*). Further, these expression events are focused at the critical junction in disease development of moderate dysplasia (CIN II) indicating a role for chromatin remodelling as part of cervical cancer development.

**Conclusion:**

We have created a valuable publically available resource for the study of gene expression in precancerous cervical lesions. Our results indicate deregulation of the chromatin remodelling complex components and its influencing factors occur in the development of CIN lesions. The increase in SWI/SNF stabilizing molecule *SMARCC1 *and other novel genes has not been previously illustrated as events in the early stages of dysplasia development and thus not only provides novel candidate markers for screening but a biological function for targeting treatment.

## Background

Cervical cancer affects approximately 500,000 women worldwide each year with highest rates in developing countries [1]. Cervical intraepithelial neoplasia (CIN) is a precursor lesion to cervical cancer and can be further subdivided into CIN I, CIN II and CIN III (mild, moderate and severe dysplasia, respectively). Most CIN I lesions spontaneously regress to normal however CIN III lesions are much more likely to progress to cervical cancer if left untreated [1]. The CIN I to CIN II junction may be critical in disease development.

Human Papillomavirus (HPV) is the recognized etiologic agent for cervical cancer however, alone it is not sufficient for invasive disease. HPV is detected in nearly all cervical cancers, 94% of CIN lesions and up to 46% of normal cervical epithelium [1]. Over 100 strains of HPV exist however HPV 16 and HPV 18 are considered highly virulent strains and account for the majority of cervical cancers [1,2].

The study of cervical cancer prevention has progressed impressively in the recent past. Widely implemented screening programs have resulted in 80% reduction of cervical cancer rates in North America within the past fifty years [3]. Cytological assessment is currently the frontline method for identifying precancerous cervical lesions however repeat evaluations can frequently be required due to low sensitivity [3]. Although vaccines against the most virulent strains of HPV have recently become available, vaccination is not an easily utilized resource for those countries most inflicted with the highest cervical cancer rates [3]. This is largely due to cost and that the administration of the vaccine occurs in three doses over six months to a pre-adolescent female population. Few countries have established programmes targeting healthcare to this population [4]. In addition, perceived social implications in developed countries regarding vaccinating girls at an early age are hindering widespread administration. Together, these social, clinical and genetic factors indicate that frontline monitoring will continue to play an important role in cervical cancer prevention and that improved methods and markers for detection are needed.

It is unclear that HPV alone is responsible for disease progression. A thorough understanding of genetic events in precancerous cervical intraepithelial neoplasia is required to both delineate important causal events in cervical cancer and to identify informative candidate biological markers. Gene expression of cervical tissue and changes in expression pattern have been the focus of several recent publications. Studies by Pérez-Plasencia et al and Shadeo et al both characterized the transcriptome of normal cervical epithelium using serial analysis of gene expression (SAGE) [5,6], Additionally, Gius et al reported changes in proproliferative/immunosuppression gene expression in CIN I lesions, as well as proangiogenic and proinvasive expression signatures that coincide with CIN II and CIN III, respectively [7].

In this study, we build upon our previous work in defining the normal cervical epithelial transcriptome and aim to identify genes differentially expressed between normal cervical epithelium and those precancerous lesions which are more apt to progress to cervical cancer if left without treatment (CIN III). In this study we have distinguished gene expression aberrations across mild/moderate dysplasia (CIN I, CIN II) in addition to CIN III and non cancerous (NC) cervical epithelium using an unbiased long serial analysis of gene expression (L-SAGE) method that simultaneously allows for the discovery of tags which map to HPV 16. In total, sixteen L-SAGE libraries were sequenced for a total of 2,481,387 tags, establishing the largest SAGE data collection for cervical tissue worldwide. Upon evaluation of expression differences between NC cervical epithelium and CIN lesions, we have identified two gene networks directly or indirectly involved in chromatin remodelling altered in expression in CIN III.

## Results

### L-SAGE Libraries from Cervical Tissue

**Figure 1:**
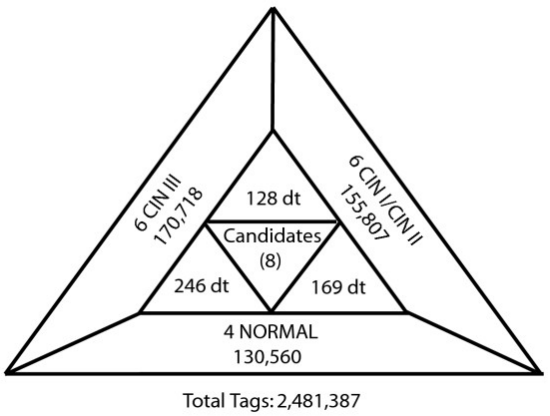
**Summary of L-SAGE library analysis.** Sixteen L-SAGE libraries were created and a total of 2,481,387 useful tags were sequenced. An average of 130,560 tags were sequenced per NC library (4), 155,807 per CIN I/CIN II library (6) and 170,718 per CIN III library (6). One-hundred and sixty-nine tags were differentially expressed when comparing NC to CIN I/CIN II, 246 between NC and CIN III and 128 tags were differentially expressed between CIN I/CIN II and CIN III. Eight candidates were identified for greatest amplitude of change between NC and CIN III and gene network most affected.

In this study 16 L-SAGE libraries were constructed and analyzed (Figure 1). Libraries N1 to N4 were made from NC cervical tissue samples, M1 to M3 from CIN I samples, M4, to M6 from CIN II samples and C1, to C6 from CIN III samples. N1, N2, C1 and C2 were mined in a preliminary study characterizing normal cervical tissue [6]. Collectively, 2,481,387 useful tags were sequenced (Figure 1). This data collection has been made publicly available at Gene Expression Omnibus, series accession number GSE7433 [8].

### Early Stage Changes

The mean tag counts were compared between NC tissue samples (libraries N1-4) and mild/moderate stage (CIN I/II) samples (libraries M1-6) in order to identify tags differentially expressed in the early stages of neoplasia. One-hundred sixty-nine tags were identified to be differentially expressed according to our selection criteria as described in Methods [see Additional file 1]. Both increased and decreased expression is observed at comparable frequencies (75 tags increased, 94 tags decreased in CIN I/II) and 138 of these tags mapped to known genes including loci encoding hypothetical proteins.

**Figure 2:**
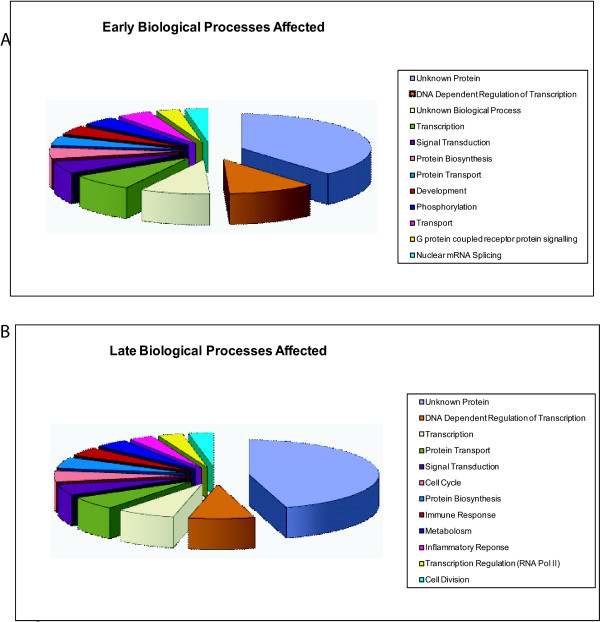
**Functional groupings of tags differentially expressed in SAGE libraries.** (A) Genes differentially expressed between NC and CIN I/CINII. (B) Genes differentially expressed between CIN I/CIN II and CIN III. Categories are as described. The Unknown function group consists of tags mapping to genes with no known function. DNA Dependent Regulation of Transcription was the function most targeted in both groups of differentially expressed tags.

The most commonly affected biological process in the early events of disease is DNA dependent regulation of transcription and transcription (11% and 8% of differentially expressed tags, as assessed by Onto-Express, respectively) (Figure 2A) [9,10].

### Late Stage Changes

The mean tag counts were compared between CINI/II tissue samples (libraries M1-6) and CIN III libraries (C1-6). This identified 128 tags differentially expressed in later stages of neoplasia [see Additional file 2]. Seventy-three tags were increased in CIN III while 55 were decreased. Overall 97 tags mapped to known genes. The major pathways affected by these genes were similar to those observed in early stage changes, DNA dependent regulation of transcription and transcription (8% and 7% as assessed by Onto-Express. respectively) (Figure 2B) [9,10]. Twelve tags mapped to the HPV 16 genome that could not be mapped to the human genome or transcripts using methods previously described [see Additional file 3]. [6]. In addition, 42 tags showed concurrent expression between all sixteen libraries [see Additional file 4].

### Pathway Analysis of Overall Changes

**Figure 3:**
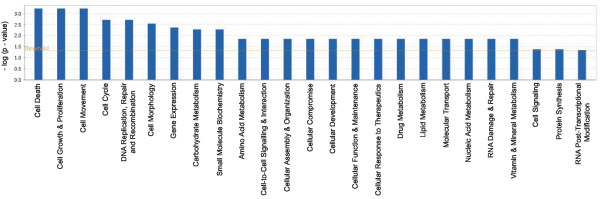
**Biological functions targeted by gene expression changes between NC and CIN III Functional categories deemed as significant by Ingenuity Pathway Analysis software, are displayed along the x-axis.** The y-axis displays the -(log) significance and the orange horizontal line denotes the cut off for significance (p-value of 0.05). The functional categories most significantly influenced include cell death, cell growth and proliferation and cell movement.

**Figure 4:**
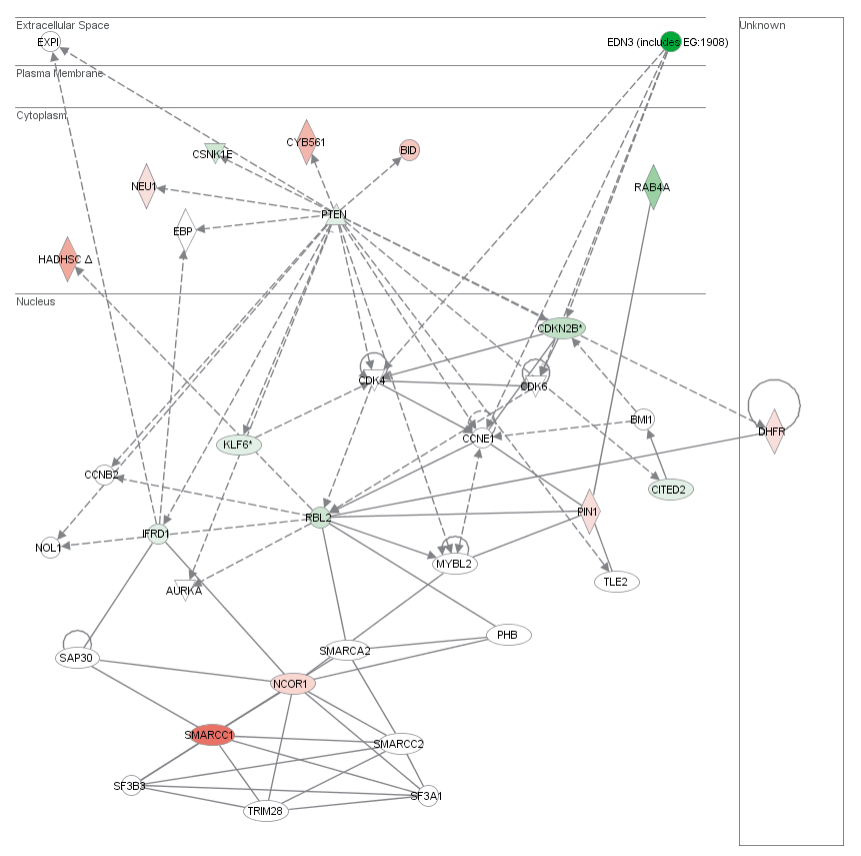
**Gene Network A.** Green denotes loss in expression in CIN III while red indicates gain. Intensity of colour signifies magnitude of change. Solid lines indicated direct interactions while dashed lines indicate indirect interactions as described by Ingenuity Pathway Systems. Functions influenced by Network A include cell cycle, cancer and cell morphology.

**Figure 5:**
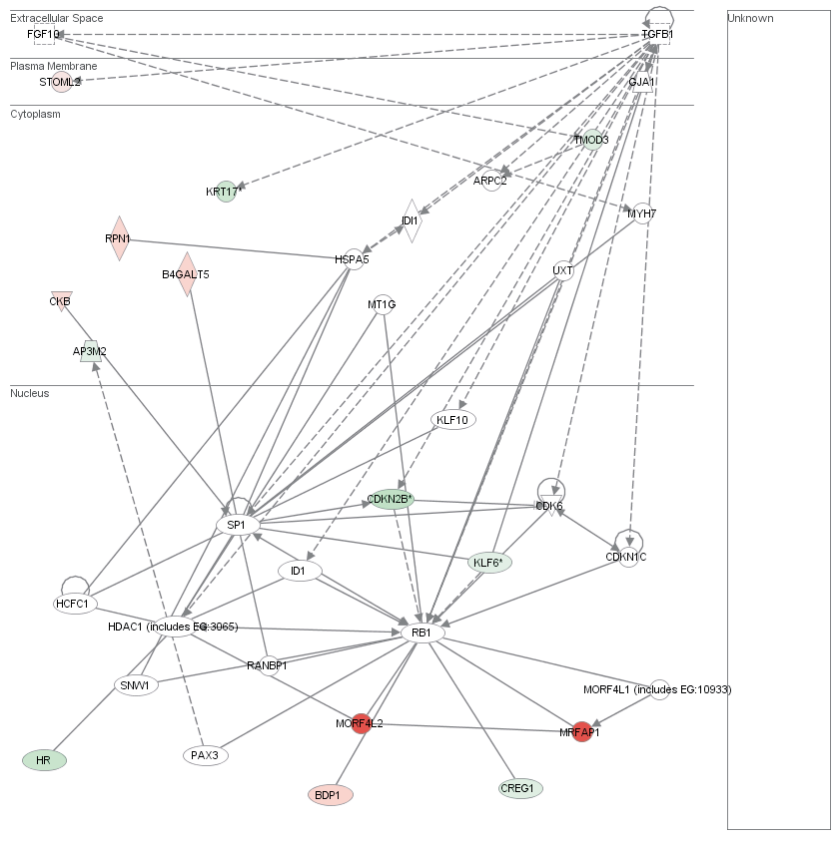
**Gene Network B.** Green denotes loss in expression in CIN III while red indicates gain. Intensity of colour signifies magnitude of change. Solid lines indicated direct interactions while dashed lines indicate indirect interactions as described by Ingenuity Pathway Systems. Functions influenced by Network B include cell cycle, gene expression and cancer specific function.

The mean score of tags in the four NC libraries were compared to the mean score of tags in the six severe neoplasia libraries (CIN III). We identified 108 tags increased in frequency in CIN III and 138 tags decreased and overall observed 246 tags differentially expressed between NC tissue and CIN III [see Additional file 5]. Two hundred and forty tags mapped to 222 unique genes. Genes differentially expressed between normal and CIN III were evaluated for gene network associations using Ingenuity Pathway Analysis [11]. Biological functions most influenced by these genes include cell death, cell growth/proliferation and cellular movement (Figure 3). The gene network with the most number of differentially expressed genes influenced cellular processes such as cell cycle and cell morphology and involved 17 of the differentially expressed genes (Table 1, Figure 4). The network with the higher magnitude gene expression changes influenced cell cycle and gene expression (Figure 5).

**Table 1:** Genes differentially expressed in Networks A and B.

Network	IPA Score*	Increased in CIN III	Decreased in CIN III
A	22	BH3 interacting domain death agonist	**cyclin-dependent kinase inhibitor 2B**
		cytochrome b-561	Cbp/p300-interacting transactivator, with
		**dihydrofolate reductase**	Glu/Asp-rich carboxy-terminal domain, 2
		**nuclear receptor co-repressor 1**	endothelin 3
		sialidase 1	interferon-related developmental regulator 1
		peptidyl-prolyl cis/trans isomerase, NIMA-	Kruppel-like factor 6
		interacting	**phosphatase and tensin homolog RAB4A**
		**SWI/SNF related, matrix associated, actin dependent regulator of chromatin, subfamily c, member 1**	**retinoblastoma-like 2 (p130)**

B	17	UDP-Gal:betaGlcNAc beta 1,4- galactosyltransferase, polypeptide 5	adaptor-related protein complex 3, mu 2 subunit
		B double prime 1, subunit of RNA polymerase III transcription initiation factor IIIB	**cyclin-dependent kinase inhibitor 2B **cellular repressor of E1A-stimulated genes 1
		creatine kinase, brain	Kruppel-like factor 6
		**mortality factor 4 like 2**	keratin 17
		**Mof4 family associated protein 1**	tropomodulin 3
		ribophorin I	
		stomatin (EPB72)-like 2	

* The score is a numerical value used to rank networks according to how relevant they are to the genes in your input dataset. Calculations are based on the hypergeometric distribution calculated via the computationally efficient Fisher's Exact Test for 2 × 2 contingency tables^8^. Gene symbols in bold denote those selected for validation in a new panel of fourteen cases.

Eight genes from these networks were selected for validation in an independent panel of cervical samples (Table 2). Cyclin-dependent kinase inhibitor 2B *(CDKN2B)*, retinoblastoma-like 2*(p130 or RBL2) *and phosphatase and tensin homolog *(PTEN) *had lower counts in the CIN III derived libraries. Conversely, Mof4 family associated protein 1 *(MRFAP1)*, mortality factor 4 like 2*(MORF4L2)*, nuclear receptor co-repressor 1 *(NCOR1)*, dihydrofolate reductase *(DHFR) *and SWI/SNF related, matrix associated, actin dependent regulator of chromatin, subfamily c, member 1*(SMARCC1) *showed higher tag counts in the CIN III relative to NC.

**Table 2:** Candidate genes in Networks A and B.

**Gene**	**P Score N vs CINIII**	NC **Mean**	**CINI/II Mean**	**CINIII Mean**	**Fold Change**
SWI/SNF related, matrix associated, actin dependent regulator of chromatin, subfamily c, member 1	2.78	1.83	7.49	20.21	11.02
Mof4 family associated protein 1	2.14	1.83	3.64	23.67	12.90
mortality factor 4 like 2	3.36	1.78	18.65	35.20	19.78
nuclear receptor co-repressor 1	2.36	12.65	38.27	42.02	3.32
dihydrofolate reductase	2.68	10.07	28.04	27.47	2.73
cyclin-dependent kinase inhibitor 2B	2.56	23.95	9.32	3.67	-6.53
retinoblastoma-like 2 (p130)	2.43	31.05	15.54	5.70	-5.45
phosphatase and tensin homolog	2.32	20.42	4.95	5.89	-3.47

**Figure 6:**
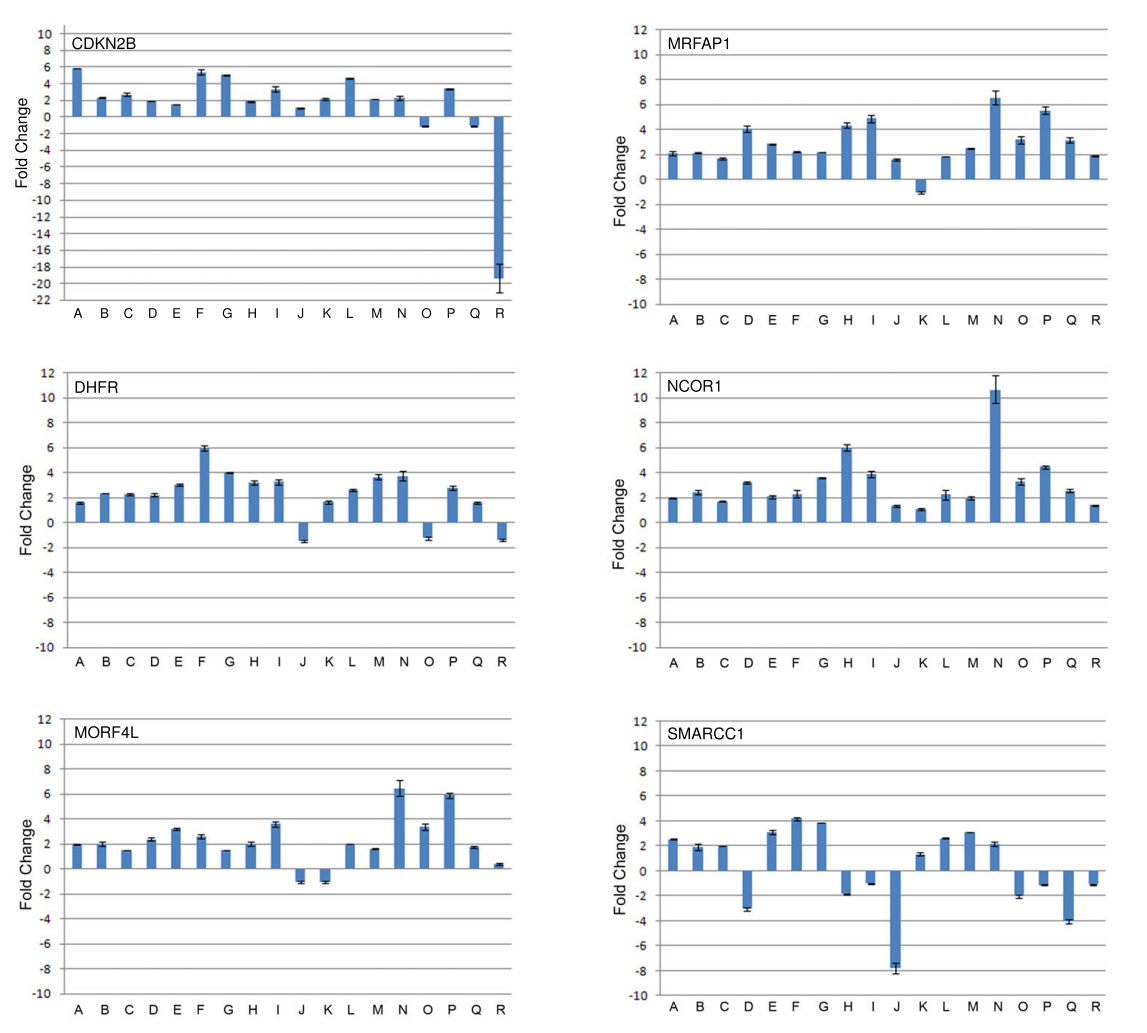
**Summary of validation panel quantitative PCR results of genes with altered expression in CIN III L-SAGE libraries.** A panel of 22 new cases was investigated in triplicate for expression changes for eight genes. Samples are indicated as follows: CIN I (A-C), CIN I/II (D), CIN II (E-J), CIN III (K-Q) and tumour (R). Zero on the Y-axis denotes mean expression levels of the respective genes in three NC cervical tissues. Expression in tumour is relative to matched normal.

The validation panel consists of 22 samples representing NC, CIN I, CIN II and CIN III in addition to a tumor sample with paired normal tissue. Quantitative analysis of expression was determined for each gene on this panel by real-time PCR using TaqMan^® ^Gene Expression Assays (Figure 6). *NCOR1 *expression increased in nine of ten CIN I/II samples ranging from 1.3–6.0 fold increase relative to NC with a statistically significant overall all trend (*p *< 0.05). *SMARCC1 *expression increase was confirmed in six CIN I/II samples and four CIN III cases (Figure 6). Similarly, we confirmed the increased expression of *DHFR *and *MRFAP1 *in the validation panel including in nine of ten CIN I/II cases and six of seven CIN III cases and the overall trend of increase for this gene was statistically significant (*p *< .05). *MORF4L2 *expression increase was confirmed in eight of ten CIN I/II and six of seven CIN III with statistically significant overall trend of increase (*p *< .05) in the validation panel. Although we were able to confirm decreased *CDKN2B *expression in the tumour, we were unable to confirm this in the earlier staged cases in the validation panel. Reduced expression of phosphatase and tensin homolog (*PTEN) *was confirmed in two CIN II and two CIN III cases [see Additional file 6].

## Discussion

The study of precancer lesions is essential in understanding the initiating events in cancer development and is of particular importance in cervical cancer as moderate and high grade lesions (CIN II and CINIII) are more likely to progress to cancer than those of low grade (CIN I). In this study, we have comprehensively evaluated gene expression changes in the precancerous stages of cervical cancer by comparing 16 cervical intraepithelial neoplasia and NC cervical specimens using the L-SAGE method of gene expression profile determination. We have sequenced a total of 2,481,387 tags, establishing the largest SAGE data collection for cervical tissue worldwide. CIN I, CIN II, CINIII as well as normal tissues are represented in this collection. In a preliminary study, we had identified Keratin 6A (KRT6A), carcinoembryonic antigen-related cell adhesion molecule 7 (CEACAM7), S100 calcium-binding protein A7 (S100A7) and small proline-rich protein 3 (SPRR33) to be highly expressed (>500 TPM) in NC cervical tissue in the N1 and N2 libraries [6]. Tags mapping to these genes are present at high levels in the additional normal libraries (N3 and N4) unique to this study with the exception of CECEAM7 which shows slightly reduced expression of 363 and 397 TPM (N3 and N4, respectively). The decreasing trend in Galectin 7 (LGALS7) and Gap junction protein A1 (GJA1) previously observed in CIN III when compared to normal mean values, continues to be true in this study (3.0 and 1.7 fold decrease in CIN III, respectively, p value not significant).

### Early and Late Events

Of the tags differentially expressed between NC and mild/moderate dysplasia (169 tags), and between mild/moderate and severe dysplasia (128 tags); 25% fewer tags were altered in later stage disease. These differentially expressed tags most frequently mapped to genes involved in DNA dependent regulation of transcription. Interestingly, tags mapping to genes with biological functions unique to late stage changes include cell cycle (5% of tags), cell division (3%), immune (4%) and inflammatory (3%) response.

### Biological Characteristics of Differentially Expressed Tags

Of the 246 tags that are altered in expression in CIN III relative to NC, cell death, cell growth and proliferation, cell movement, cell cycle and DNA replication, recombination and repair are the top five biological functions. These functions encompass characteristics frequently described as the hallmarks of cancer [12].

Tags mapping to seven unique genes showed a greater than ten fold increase in expression in CIN III libraries. A tag mapping to SEC13 homolog (*SEC13L1*) showed the greatest change as it was not present in any one of the four NC libraries. However, an average of 20 TPM were observed in CIN III libraries. Additional tags with greater than ten fold change mapped to *MORF4L2*, *MRFAP1*, WD repeat domain 18(*WDR18*), *SMARCC1*, eukaryotic translation elongation factor 1 gamma (*EEF1G*) and G protein-coupled receptor 180 (*GPR180*). *SMARCC1 *is a component of the chromatin remodeling complex SWI/SNF while both MRFAP1 and MORF4L2 belong to the MGF/MORF family of transcription factors involved in growth, cell senescence and are implicated indirectly in chromatin remodeling [13-18]. *WDR18 *is a member of the WD repeat protein family. Members of this gene family are involved in a variety of cellular processes including cell cycle progression, signal transduction, apoptosis, and gene regulation [19]. Tags mapping to this gene varied in counts between NC and CIN III by greater than twelve fold increase in the latter subgroup. *EEF1G *is a subunit of the elongation factor-1 complex involved in translation and *GPR180 *contains transmembrane domains and may play a role in vascular remodelling [20]. It is interesting to note that both homeobox B7 (*HOXB7) *and BH3 interacting domain death agonist (*BID*) are also increased in CIN III although to a lesser magnitude (seven and five fold, respectively). Many of the described genes may have the potential to influence processes characteristic of epithelial cancers such as, apoptosis (*BID*), angiogenesis (*GPR180*), proliferation (*WDR18*), and transcription influencing events (*SMARCC1*, *MRFAP1*, *MORFL2*). Functional assays are required to delineate their biological function in CIN and cervical cancer.

### Network Analysis

Upon further investigation of the 246 differentially expressed tags between NC and CIN III, 240 mapped to 222 unique genes. When analyzed for gene network relationships, many of the genes targeted pathways influencing properties such as cell cycle, cell morphology, cancer related events and gene expression. In this study, we have identified a group of 28 genes that fall into two gene networks encompassing these properties. The first network (A) contains 15 of the differentially expressed genes while the second network (B) includes 13 of the 222 genes. Only cyclin-dependent kinase inhibitor 2b (CDKN2B) and kruppel-like factor 6 (KLF6) overlap between Network A and B. Network B includes three of the seven tags showing greater than 11 fold change and one of the five tags showing more than 20 fold decrease in NC when compared to CIN III. Genes from both networks were selected for validation in a new cervical tissue panel.

### Network A

Cell cycle, cancer and cell morphology are the top functions influenced by genes in Network A. Sixteen genes differentially expressed between NC and CIN III are involved in this network including *RBL2/p130*, *SMARCC1*, *NCOR1*, *PTEN*, *DHFR *and *CDKN2B*.

*RBL2/p130 *is one of three members of the Retinoblastoma (Rb) gene family, others include *RBL1/p107 *and *RB1 *[21]. This family of proteins regulate cell cycle through the G1/S phase by sequestering E2F transcriptional regulators. Loss and mutations in *RBL2/p130 *have been linked to various cancers [22,23]. E7 from high risk HPV strains targets all member of the Rb family including RBL2/p130, for degradation thus releasing sequestered E2F and allowing for passage through the G1/S phase [24,25]. Zhang et al found that low risk HPV 6 E7 also has the capacity to bind RBL2/p130 although not with as high affinity and did not bind to the other member of the Rb family [25]. We observed a progressive reduction in *RBL2/p130 *transcripts from NC to mild/moderate dysplasia and severe dysplasia suggesting that a reduction in *RBL2/p130 *may also be regulated at the transcriptional level in cervical preneoplasia and may be the first Rb family gene event in the development of cervical intraepithelial neoplasia.

*SMARCC1 *is a member of the SWI/SNF family of genes. Members of this evolutionarily conserved gene family are proposed to regulate gene specific transcription with downstream effects in cell cycle progression [26]. SMARCC1 has recently been reported to directly interact with components of the ATP-dependent SWI/SNF chromatin remodelling complex including, SWI/SNF-related, matrix-associated, actin-dependent regulator of chromatin A4 (BRG1), BRG1-associated factor A (BAF60a) and SWI/SNF-related, matrix-associated, actin-dependent regulator of chromatin B1 (SNF5) [15]. The SWI/SNF chromatin remodelling complex consists of approximately ten components and is thought to regulate gene transcription by altering the surrounding chromatin structure. This complex can recruit both histone acetyltransferases (HATs) and histone deacetyltransferases (HDACs) in a gene specific manner thus influencing gene expression [27]. The SWI/SNF chromatin remodelling complex also has been implicated in hormone receptor signalling and growth [16]. SMARCC1 was characterized as a key regulator of core complex components (BRG1, BAF60a and SNF5) by positively influencing the stabilization of the complex proteins as opposed to regulation of their transcription [15]. In our study, there was no expression change in these core components. In contrast, we observed an 11 fold increase in tags mapping to SMARCC1 in CIN III libraries when compared to normal libraries suggesting that SWI/SNF chromatin remodelling complex stability may have a role in severe dysplasia development.

*DHFR *is involved in folate metabolism in eukaryotes and is essential for purine and thymidylate biosynthesis and thus DNA replication [28]. We observed over a 250% increase in both mild/moderate and severe dysplasia as compared to normal, indicating that this event is present in very early stage lesions. It has recently been reported that *DHFR *is subjected to RB mediated repression via SWI/SNF chromatin remodelling activity [29]. The increase in *DHFR *observed is in concordance with the expected downstream effects of decreased RB gene family repression.

*NCOR1 *is involved in transcription repression via chromatin condensation. It has been found to physically interact with members of the SWI/SNF complex, including SMARCC1 and core component BRG1 [30]. We observed greater than 300% increase in NCOR1 in severe and mild/moderate dysplasia relative to normal suggesting an increase in NCOR1 occurs very early in disease development (CIN II/CIN I).

Phosphatase and tensin homolog (*PTEN)*, an established tumour suppressor gene, functions through the AKT/PKB signalling pathway [31]. We found *PTEN *to decrease in expression in CIN II/CIN I libraries by >3 fold compared to normal indicating that this may be an early event. *CDKN2B *negatively regulates CDK4 and CDK6 [32]. We found *CDKN2B *decreased in CIN III libraries by nearly 3.5 fold when compared to NC. Decreased expression of *CDKN2B *has not been found due to copy number loss or mutation in cervical cancer [32].

Genes from Network A were selected for validation in a new 14 sample set consisting NC, CIN I, CIN II and CIN III cervix tissue and one tumour and normal pair. A similar pattern of fold change is present in this panel for *DHFR*, *SMARCC1 *and *NCOR1 *in all three subgroups. Specifically, increased expression is observed in all three CIN II and two CIN III in the new cervical tissue panel, validating the disruption of genes involved in chromatin remodelling.

For PTEN, we observed a decrease in one of the three CIN III cases investigated which is consistent with Lee et al who found PTEN to be reduced in protein expression in only 10% of CIN III cervical cases and 18% of cervical cancers [33]. We also found CDKN2B to be over expressed in all CIN I, two CIN II and one CIN III however, greater than four fold decrease in cervical cancer (T1) was observed. It has been reported that cervical cancers and CIN III lesion have elevated levels of CDKN2B (84% and 79%, respectively) [34].

### Network B

Fourteen genes differentially expressed between normal and CIN III targeted Network B including *MRFAP1*, *MORFL2 *and *CDKN2B*. Top functions influenced by Network B include cancer related factors, cell cycle and gene expression. The previously discussed gene *CDKN2B *overlaps with this network.

*MRFAP1 *binds with strong affinity to mortality factor 4 like 1 (*MRG15*) [17]. *MRG15 *is a member of the MRG/MORF family of genes. All members contain a MRG domain which has the capacity to bind multiple transcriptional regulators [18]. Members of this gene family are suggested to be involved in embryonic development, cell proliferation and senescence [35]. MRG15 is a component of the MRG associated factors 1 and 2 complexes (*MAF1*, *MAF2*, respectively) and thus has a putative role in chromatin remodelling [36]. MRG15 has been shown to specifically associate with MRFAP1 in MAF1, tumour suppressor RB and the mammalian co repressor complex, mSin3A [14,35]. We observed a 12.9 fold increase of *MRFAP1 *in CIN III libraries when compared to NC libraries. Notably, the majority of this increase occurs between mild/moderate and severe dysplasia. Although the biological mechanisms of MRFAP1 and MRG15 interaction has yet to be described, it is plausible that the relationship may influence the essential role MRG15 plays in chromatin remodelling.

We observed a 20 fold increase of *MORF4L2 *in CIN III libraries. Like *MRG15*, *MORF4L2 *also belongs to the MRG/MORF transcriptional regulator family of genes involved in senescence and cell growth [37]. Although the two genes share nearly 90% similarity, *MORF4L2 *is unique to vertebrates whereas MRG15 is evolutionarily conserved [14,37]. Similarly to MRG15, MORF4L2 directly interacts with MRFAP1 and the mSin3A co repressor complex, components of which include HDAC1 and HDCA2 and are involved in chromatin remodelling [13,14]. MORF4L2 is reported to have both repressive and stimulatory activity in transcription regulation of well known cell cycle regulator, v-myb myeloblastosis viral oncogene homolog (avian)-like 2 (*MYBL2) *[14,38].

*MRFAP1 *and *MORFL2 *were selected from Network B for validation in 22 new samples as described above. In both cases, six of seven CIN III samples showed the greatest increase in expression (1.8 – 6.6 fold increase) and the overall increasing trend was found to be statistically significant (*p *value .033 and .017, respectively). We observed a 2.0–2.5 fold increase in expression in cervical cancer (T1). Together, these results validate the differential expression again supporting a potential role of chromatin remodelling in cervical cancer progression.

**Figure 7:**
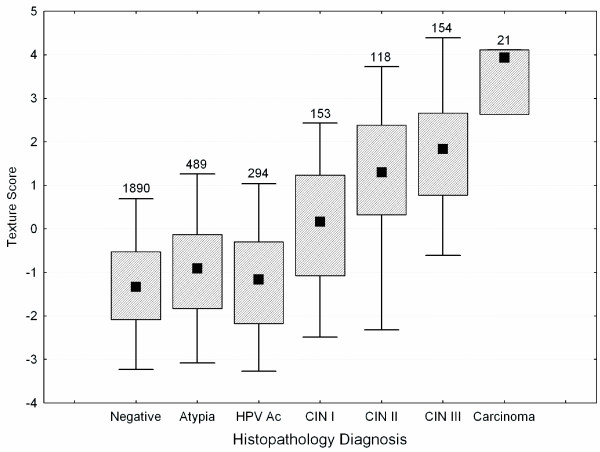
**An updated box plot from Guillaud et al.** showing the correlation between a linear discriminant function score (Texture Score) based entirely on only texture phenotype features measured of the nuclei in formalin fixed quantitatively stained sections of biopsied tissue [39]. The error bars represent the 5^th ^and 95^th ^percentiles; the box represents the central 50^th ^percentile and the solid square the mean of the distribution of the scores for measured sections with the noted histopathological diagnosis. The numbers over the boxes are the number of samples measured for the specific diagnosis.

This differential expression in the chromatin remodelling genes could result in changes in the organization of the DNA. If these changes are large enough they could be detectable. Previously, we quantified changes in nuclear texture with increasing grade of CIN [39]. These measured phenotype changes, as seen in Figure 7 (an updated graph of the data), begin with CIN I and are more prominent in the high grades of CIN (II, III) and cancer [39]. This appears to correlate CIS with the novel observations presented in the present study with respect to the increased expression of chromatin remodelling complex components indicating that these expression changes result in an alteration of the cell/nuclear phenotype.

## Conclusion

Events in expression change involving genes in Network A and Network B (*DHFR*, *MORFL2*, *MRFAP1*, *NCOR1*, and *SMARCC1*) occur at or before the stage of moderate dysplasia (CIN II). These events are maintained, although at a reduced frequency, in CIN III suggesting a role in intermediate events prior to severe dysplasia that may be an important stepping stone in disease progression. Genomic instability is characteristic of CIN III lesions and cervical cancer, as such the non-maintenance of such aberrations in later stages may be due to the masking the of initial events by additional changes acquired in severe dysplasia [3,40,41].

Deregulation of chromatin remodelling functions has analogous effects to mutation in DNA repair components in that the repercussions can be genome wide. It is interesting that evidentially two deregulated genes from both networks investigated are associated with chromatin remodelling (*SMARCC1*, *NCOR1*, *MRFAP1*, *MORF4L2*) and such disruptions are targeted to the critical stage of moderate dysplasia. The increase in SWI/SNF stabilizing molecule *SMARCC1 *and other novel genes has not been previously illustrated as events in the early stages of dysplasia development thus providing not only novel candidate markers for screening but a biological function for targeting treatment. Together, our results suggest altered expression events in chromatin remodelling complex components and influencing factors occur in precancerous cervical intraepithelial neoplasia. Future investigation on protein DNA interaction will be necessary to further elucidate the precise role of chromatin remodelling in cervical cancer progression.

## Methods

### Sample Selection and Collection

Specimens were collected as previously described [6], immediately prior to the LEEP (Loop electrosurgical excision procedure) using bite biopsy targeting a small portion of the affected epithelium. These specimens were collected with patient consent and University of British Columbia (UBC) British Columbia Cancer Agency (BCCA) Research Ethics Board approval at the Vancouver General Hospital Women's Clinic at the Vancouver Hospital & Health Science Centre (VHHSC). Anonymized cases were assessed by cervical cancer pathologists at BCCA and were selected without prior knowledge of HPV status. Specimens N1to N4 in this study were observed to be non cancerous squamous epithelia whereas C1toC6 were identified as high grade dysplasia or CIN III. Specimens M1to M3 were assessed as CIN I while M4to M6 as CIN II. Detail description of LEEP cone specimens are listed in Additional file 7. All samples were stored in a RNA preservation solution (RNA *later*, Ambion) within 2 minutes of retrieval and then placed in storage at -80°C.

### L-SAGE

The biopsies were homogenised in Lysis Binding buffer (100 mM Tris-HCl, pH7.5, 500 mM LiCl, 10 mM EDTA, pH 8.0, 1% LiDS, 5 mM dithiothreitol). Long SAGE libraries were constructed using the L-SAGE kit (Invitrogen, Ontario, Canada) and sequenced to an average depth of 155,000 tags allowing for deep sampling of the transcriptome without an initial restriction to known genes [42-44].

### Analysis

Raw tag count data for each SAGE library was normalized to tags per million (TPM) to facilitate comparison between libraries. Each sequence tag was then mapped to genes using the August 1 2006 version of SAGEGenie [45]. CIN I and CIN II libraries were grouped in analysis (CIN I/CIN II or mild/moderate dysplasia). Subsequently, those tags detected at a level of 20 TPM in at least one of the NC libraries, CINI/CINII, and CINIII groups were retained for differential expression analysis. To determine differential expression between the three groups, a combination of a two-fold difference in means as well as a permutation score ≥ 1.96 (corresponding to an unadjusted p-value of 0.05) was used [46]. Briefly, the permutation test used in this analysis examines the statistical significance of the observed differences in means between two groups in comparison to the average and standard deviation of difference of 10,000 random permutations of the data.

### cDNA synthesis

A validation panel of 12 cervical specimens (independent from those used in the SAGE analysis) were assembled: normal (Na, Nb, Nc), CIN I (CIa, CIb, CIc), CIN II (CIIa, CIIb, CIIc) and CIN III (CIIIa, CIIIb, CIIIc). Specimens were collected and stored as described above. RNA was isolated as previously described [6]. In addition, cervix squamous cell carcinoma (T1) and adjacent normal tissue (N1) total RNA sample set was purchased (Ambion, #7276). All cDNA was generated from 200 ng of total RNA using the High Capacity TaqMan Reverse Transcription Reagents (Applied Biosystems, Foster City, CA).

### Realtime PCR Analysis

The expression levels of individual genes were analyzed by real-time PCR using TaqMan^® ^Gene Expression Assays in triplicate in a new panel of 22 cases using the ABI 7500 Real-Time PCR System (Applied Biosystems). Taqman probes (Assay IDs) include *18S *(Hs99999901_s1), *MORF4L2 *(Hs0020211_m1), *MRFAP1 *(Hs00738144_g1), *SMARCC1 *(Hs00268265_m1), *NCOR1 *(Hs00196920_m1), *DHFR *(Hs0075822_s1), *PTEN *(Hs00829813_s1), *RBL2 *(Hs00180562_m1) and *CDKN2B *(Hs00793225_m1). The relative quantification of the target genes in CIN I, CIN II and CIN III samples compared to the average Ct of NC samples was performed using the established 2^-ΔΔCt ^method (Applied Biosystems, Relative Quantitation Of Gene Expression, ABI PRISM 7700 Sequence Detection System: User Bulletin #2). Gene expression is normalized to *18S*. The relative quantification values were then plotted, a ratio of one indicating no change with respect to NC cervical tissue. A single squamous cervical carcinoma sample and adjacent normal tissue were also compared using this method (T1 and N1, respectively).

## Abbreviations

Cervical Intraepithelial Neoplasia (CIN), Human Papillomavirus (HPV), Serial Analysis of Gene Expression (SAGE), Tags per million (TPM)

## Authors' contributions

AS designed, organized and performed this study and wrote the manuscript. RC performed data analysis. AP performed gene specific expression assays. KL contributed to the project design, directed library construction and edited the manuscript. DM, TE, DN, JM, RR, MF and MG were responsible for clinical diagnosis, sample acquisition and pathology assessment. WL and CM are shared principle investigators of this study. All authors read and approved the final draft of this manuscript.

## Supplementary Material

Additional file 1 Genes differentially expressed in the early stages of neoplasia. Scales tags differentially expressed between normal and CIN I/II. Click here for file

Additional file 2 Genes differentially expressed in the later stages of neoplasia. Scales tags differentially expressed between CIN I/II and CIN III. Click here for file

Additional file 3 Tags mapping to the HPV 16 genome. Tags and scaled tag counts described. Click here for file

Additional file 4 Genes concurrently expressed in all 16 libraries. Scales tags expressed at similar levels in all 16 libraries. Click here for file

Additional file 5 Genes differentially expressed between normal cervical epithelium and severe dysplasia. Scales tags differentially expressed between normal and CIN III. Click here for file

Additional file 6 Validation panel quantitative PCR results of genes with altered expression in CIN III L-SAGE libraries. A panel of 22 new cases was investigated in triplicate for expression changes for eight genes. Samples are indicated as follows: CIN I (A-C), CIN I/II (D), CIN II (E-J), CIN III (K-Q) and tumour (R). Zero on the Y-axis denotes mean expression levels of the respective genes in three NC cervical tissues. Expression in tumour is relative to matched normal. Click here for file

Additional file 7 Pathology on all cases. Pathology on all cases used as described by pathologist at the British Columbia Cancer Agency. Click here for file
