# Supplementary material for: Up regulation in gene expression of chromatin remodelling factors in cervical intraepithelial neoplasia
Source: BMC Genomics. 2008 Feb 4;9:64. doi: 10.1186/1471-2164-9-64 (PMC2277413; doi:10.1186/1471-2164-9-64)
Supplement: Additional file 1 — Genes differentially expressed in the early stages of neoplasia. Scales tags differentially expressed between normal and CIN I/II. [file 1471-2164-9-64-S1.doc]

**Supplemental Table 1**

All differentially expressed tags between Normal and Moderate Dysplasia

| Tag | Symbol | NC_Mean | CINIAndII_Mean | CINIII_Mean | Fold Change | PS_NC vs CINIAndII |
| --- | --- | --- | --- | --- | --- | --- |
| GACCTGTGAGAGGCACT | KIAA1324 | 1.78 | 23.50 | 27.05 | 13.20 | 2.08 |
| GAAAATTGTCACTGACT | TMEM57 | 1.83 | 23.95 | 16.61 | 13.06 | 2.62 |
| GCAGCCTGGACGTGGGG | KIAA0683 | 1.78 | 22.59 | 20.86 | 12.69 | 2.05 |
| AAGTTGGTGCTAATAAA | AMZ2 | 1.79 | 21.96 | 16.24 | 12.27 | 2.21 |
| ACTGCGAGGACGCCGCA | DPM3 | 1.78 | 21.42 | 26.12 | 12.04 | 2.00 |
| TCTTTGGGTGGGGGGGA | HSPA5 | 2.36 | 21.84 | 11.83 | 9.25 | 3.10 |
| GAAGGCATCTTCATCAA | SDF2L1 | 5.50 | 48.93 | 35.70 | 8.89 | 2.76 |
| TGGGAAAACTCCGCCTT | CYB561 | 3.62 | 26.42 | 21.56 | 7.29 | 2.59 |
| AGGACAAACCCCAATGC | GMDS | 5.36 | 38.91 | 58.25 | 7.26 | 2.23 |
| TCAGAACAGTCCAGACT | GRSF1 | 3.62 | 26.04 | 8.00 | 7.18 | 2.90 |
| CGCGTGCACACGGGCCT | ZBTB7A | 3.57 | 23.83 | 8.55 | 6.68 | 2.58 |
| TGCGGCTGGTTTTGGAC | DCTN1 | 3.62 | 23.30 | 35.95 | 6.43 | 2.07 |
| ACCTTGTGCCAGTCCTG | ST6GALNAC4 | 3.67 | 21.93 | 27.25 | 5.98 | 1.98 |
| TTGACCAGGCTGGTCTT | LOC56902 | 3.57 | 21.26 | 11.56 | 5.96 | 2.79 |
| AAAATGATATGAGATTC | SCNN1G | 7.19 | 42.58 | 29.24 | 5.92 | 1.98 |
| GCTCTTTCATATAGGAC | EVI1 | 3.57 | 20.59 | 10.88 | 5.77 | 2.18 |
| GTGGCGGGTGCCTGTAG | SORBS2 | 3.61 | 20.83 | 16.03 | 5.76 | 2.10 |
| TAGCTGCCTTTGTTACT | DVL2 | 5.35 | 30.55 | 30.08 | 5.71 | 2.28 |
| GTGGGCCAGGACGGCCA | GGT1 | 3.61 | 20.37 | 10.59 | 5.64 | 2.12 |
| AGTGCAGGGAGAAGGGC | IGL@ | 141.94 | 779.63 | 304.58 | 5.49 | 2.25 |
| ATCCGTGCCCTTGCTTC | CALM3 | 5.40 | 29.49 | 15.37 | 5.46 | 2.00 |
| TCATCTGGAATAATACT | CHFR | 4.15 | 22.35 | 3.23 | 5.38 | 2.26 |
| TCTTCCTTGGAGCTGTG | MGC13057 | 11.53 | 60.34 | 51.34 | 5.23 | 2.02 |
| TCCCCCGTGCACGGTTC | HOXB5 | 4.20 | 21.12 | 35.82 | 5.03 | 2.68 |
| CCCAACCCCTGTGGCCG | ATN1 | 4.15 | 20.23 | 17.74 | 4.87 | 2.80 |
| GGAATATGCAGAATTTC | CRLS1 | 20.43 | 97.91 | 84.44 | 4.79 | 1.99 |
| ACGAGCTGGAGCAGATC | RABL4 | 4.72 | 21.97 | 18.17 | 4.65 | 2.69 |
| AAAGCCAAGAAGAAGAA | ETFB | 5.41 | 25.16 | 22.20 | 4.65 | 2.08 |
| TGGAGCGCTACCGACAG | MRPL55 | 7.77 | 35.72 | 25.99 | 4.60 | 3.14 |
| CTCCCTTTTACATTCTG | HOXB7 | 5.46 | 24.98 | 38.39 | 4.58 | 2.10 |
| CCCGCCCCCGCCTTCCC | TOMM40 | 5.41 | 24.42 | 23.09 | 4.51 | 2.08 |
| TCATTTATAAATTTTCT | BDP1 | 6.56 | 25.90 | 24.03 | 3.95 | 2.48 |
| GATCTGTTCCTCTGTGC | GLE1L | 5.40 | 21.12 | 15.13 | 3.91 | 2.16 |
| GACAGTGACGCAAGGAC | ZNF593 | 16.26 | 60.11 | 44.10 | 3.70 | 2.21 |
| GAGTGGAGAGTTTATTC | USP9X | 10.92 | 38.39 | 40.66 | 3.52 | 2.80 |
| TAAAATTTGTACAAAAA | EIF4B | 11.95 | 41.60 | 36.91 | 3.48 | 2.44 |
| GAGAAGACTTCTTTGCA | PREP | 9.03 | 31.32 | 27.84 | 3.47 | 2.47 |
| TCTGCATCTTGGACGCC | ALDH1L1 | 10.17 | 33.73 | 59.73 | 3.32 | 2.38 |
| AGAGACAAGTCTCTTAG | RRBP1 | 13.28 | 42.56 | 52.65 | 3.20 | 2.09 |
| GACTGTTGCTGCTCCCT | SLC44A1 | 8.91 | 28.27 | 29.41 | 3.17 | 2.33 |
| GTTCATAGGTCATAGAA | EXOC4 | 7.18 | 22.60 | 4.96 | 3.15 | 2.46 |
| TAAATAAGGAAAAAATT |  | 7.82 | 23.60 | 24.17 | 3.02 | 2.66 |
| CACTGTGCCTTCAAAAT | CPD | 8.87 | 25.45 | 26.02 | 2.87 | 2.07 |
| CAAGCCCTGCTGTGTGG | C16orf34 | 18.57 | 53.31 | 59.63 | 2.87 | 2.42 |
| GGGAAACAGGTGCAAAC | C10orf54 | 19.73 | 56.58 | 41.91 | 2.87 | 2.26 |
| TCTACTGTTAGGTGAGG | NDRG3 | 11.91 | 33.87 | 25.65 | 2.84 | 2.37 |
| GCTAGGTATTTCTAATG | TES | 9.57 | 26.85 | 23.15 | 2.81 | 2.87 |
| TGCAGAACGGCCCCTTG | MRPS2 | 17.42 | 46.98 | 30.58 | 2.70 | 2.37 |
| CCGCCTCCGGGAATGAG | SNRPN | 19.26 | 50.67 | 41.35 | 2.63 | 2.09 |
| CTGTACTAGGTGCTGAA | RUSC1 | 12.60 | 32.76 | 50.18 | 2.60 | 2.30 |
| CCTGTACCCCAGATGGG | HMG20B | 18.53 | 47.83 | 42.43 | 2.58 | 2.69 |
| ATGTCCAATTTTGTTGT | SUCLG2 | 9.60 | 24.37 | 14.69 | 2.54 | 2.65 |
| TACATCCAGTGAGGGTT | VPS35 | 9.60 | 23.72 | 25.47 | 2.47 | 2.12 |
| AGGAGGGTGGGGGAGGG | LMNB1 | 11.95 | 29.50 | 31.23 | 2.47 | 2.29 |
| TTCAGAGAAACTTCTCT | WDR89 | 11.39 | 27.73 | 22.87 | 2.43 | 2.19 |
| ATCTATAAATCAGTGCT |  | 36.14 | 87.86 | 58.05 | 2.43 | 2.00 |
| CTATTTTTAATACCTGG | TMC5 | 8.34 | 20.12 | 27.96 | 2.41 | 1.98 |
| CTAGTCACTTCCTGCCC | RAB34 | 23.98 | 57.57 | 45.68 | 2.40 | 2.91 |
| GAGCATAATAAAATTAA | WASL | 14.90 | 34.99 | 25.66 | 2.35 | 2.42 |
| TAAGGATTTTTCATCTG | TMED5 | 8.97 | 20.92 | 12.07 | 2.33 | 2.00 |
| TCCCTGGCAGAGGGCTT | CRIP2 | 63.13 | 146.23 | 173.31 | 2.32 | 1.97 |
| CCACACACCGTCTCTCG | SAMM50 | 13.16 | 29.98 | 33.93 | 2.28 | 2.39 |
| CCAAGGAATGGAATTTC | LOC130074 | 12.64 | 28.56 | 18.67 | 2.26 | 1.99 |
| AAGGTGCCTCCATCTCT | UBL7 | 16.84 | 37.50 | 25.19 | 2.23 | 2.26 |
| CACTTCAAGGGCAGCCT | LY6E | 133.95 | 298.00 | 287.38 | 2.22 | 2.23 |
| CTGGGACTGACAGCCTG | LSM4 | 36.42 | 80.83 | 95.86 | 2.22 | 1.96 |
| ATTTTTGCCCTGCGTCC | RXRB | 20.32 | 45.05 | 45.60 | 2.22 | 1.99 |
| TGGAGAGCAACTTGGGA | AHCYL1 | 30.57 | 67.03 | 78.40 | 2.19 | 2.61 |
| CTACCCGGTATGACTGG | EDG4 | 13.17 | 27.74 | 29.95 | 2.11 | 1.99 |
| GATTTTTAAAATTAGTA | RALBP1 | 27.79 | 58.23 | 45.75 | 2.10 | 1.98 |
| TGCAGGTGGCACAGGTC | HYI | 13.71 | 28.70 | 31.49 | 2.09 | 2.03 |
| TGTGGTGGTGTTTTTTG | CASC3 | 16.84 | 34.84 | 25.47 | 2.07 | 2.79 |
| TGGACAGTGCACGTGCC | EYA2 | 26.19 | 53.95 | 82.24 | 2.06 | 2.24 |
| TTCATTAATTCATATTG | HNRPC | 20.32 | 41.82 | 42.49 | 2.06 | 2.41 |
| GATGTTAATTGAGAGCC | USP11 | 17.40 | 35.14 | 26.35 | 2.02 | 2.77 |
| GGCAGAGGCTCCGGGAG | ZFP36L1 | 22.72 | 11.28 | 15.07 | -2.01 | 2.04 |
| AGATGAGATGACCACCA | KLF6 | 33.38 | 16.55 | 13.44 | -2.02 | 1.99 |
| CTTATGTAGATATAAAA | NDUFB6 | 38.33 | 18.93 | 17.44 | -2.03 | 2.06 |
| CTACTCTTCTAAGTCTT | MALAT1 | 29.24 | 14.30 | 21.02 | -2.05 | 2.13 |
| TCCCTCTCAGCAGACAA | COMMD4 | 23.26 | 11.35 | 13.86 | -2.05 | 1.97 |
| CCTGTAATTCCAGCTAC | ATPBD1B | 29.92 | 14.59 | 23.72 | -2.05 | 2.15 |
| CCTGTGTGCATACCCAA | ARHGAP21 | 21.52 | 10.37 | 16.11 | -2.08 | 2.00 |
| TTTAATGATAGCTTGCT | SRP14 | 38.22 | 18.24 | 21.25 | -2.10 | 2.04 |
| GACTGAGCTTGTGGTGT | TARDBP | 103.70 | 49.42 | 66.10 | -2.10 | 2.36 |
| TTGCTATTTATGTACCT | RPL9 | 43.68 | 20.71 | 56.81 | -2.11 | 2.86 |
| TATTTTATTTGTGTATC | P2RY5 | 109.08 | 51.34 | 26.00 | -2.12 | 2.65 |
| AGCCACCGTGCCCAGCC | SGEF | 39.88 | 18.56 | 17.34 | -2.15 | 1.98 |
| AATCTTGCAAAAAAAAA | DHRS7 | 24.50 | 11.37 | 13.22 | -2.15 | 2.12 |
| GTGAATGTATGTTTCTG | EPB41L3 | 82.29 | 37.38 | 19.60 | -2.20 | 2.13 |
| GCATTCTCAGCTTAGAC | ETF1 | 41.20 | 18.48 | 22.89 | -2.23 | 2.31 |
| AAGCTATAGCTTGCTGA |  | 28.12 | 12.36 | 6.42 | -2.28 | 1.96 |
| CTATGTGTTACGTGTTT | DHX9 | 72.62 | 31.60 | 56.47 | -2.30 | 2.07 |
| GTTAAGATTTTTTTTTT | ARHGEF11 | 100.02 | 43.25 | 45.35 | -2.31 | 2.22 |
| CCTCAGCCCGCCTTCAG | GRB7 | 27.39 | 11.84 | 12.54 | -2.31 | 2.42 |
| CACATTAGGTGTTTTCA | RSNL2 | 36.32 | 15.39 | 16.75 | -2.36 | 2.01 |
| TAAAATAAGGGATCAAT | MAFB | 34.09 | 13.98 | 14.86 | -2.44 | 2.36 |
| TACATATCTGTTTAGTT | SMC6 | 26.91 | 11.01 | 9.01 | -2.45 | 2.28 |
| GTGTATATGTGCCACAT | LOC643738 | 24.56 | 10.04 | 13.84 | -2.45 | 2.72 |
| GTTTTGTACAGAAATAA | TBC1D10A | 21.48 | 8.77 | 11.02 | -2.45 | 2.12 |
| TGGGAGCCCTGTCCTCA | FAM102A | 74.62 | 30.29 | 35.28 | -2.46 | 1.98 |
| AGTAAGGTGGCTTTGAT | C4orf27 | 24.50 | 9.94 | 7.97 | -2.46 | 2.55 |
| CCTAACTCTGTACCGTT | APBB3 | 25.12 | 10.13 | 9.19 | -2.48 | 2.22 |
| CCACTGCACTCCGGCCT | RIOK3 | 41.70 | 16.80 | 24.82 | -2.48 | 2.35 |
| ACTCAAAGACCCATTGG | CEBPG | 24.56 | 9.79 | 17.00 | -2.51 | 2.16 |
| CAGGGGCTTATCTGTCT | OBFC1 | 68.45 | 27.01 | 44.17 | -2.53 | 2.23 |
| GCTGGATGCGGATATTT | C9orf3 | 80.11 | 31.36 | 33.91 | -2.55 | 2.46 |
| AAAGAGAAAAAAAAAAA | CNOT1 | 32.23 | 12.52 | 15.70 | -2.57 | 2.29 |
| TCCAGACAGCGGGGACC | EHD1 | 20.37 | 7.68 | 12.07 | -2.65 | 2.14 |
| TAATTTGATTTTGGAAT | UBE2G1 | 138.00 | 51.43 | 73.36 | -2.68 | 2.00 |
| TAACTTTGCCATCAGTT | WNK1 | 38.15 | 14.20 | 10.20 | -2.69 | 2.40 |
| GTGAAACCCCATCTCTT | ZFP90 | 31.64 | 11.71 | 20.09 | -2.70 | 2.03 |
| GGCACTTATGAGGACAA | ULK3 | 25.05 | 8.99 | 11.97 | -2.79 | 2.36 |
| GATTGAACCTCATCAAT | SC4MOL | 36.40 | 13.00 | 14.90 | -2.80 | 2.19 |
| CTCCTGGGCGCCCACGC | CALML3 | 414.33 | 143.68 | 109.23 | -2.88 | 2.21 |
| TCAGTGACCAGATTTGT | PRKCH | 20.98 | 7.22 | 8.07 | -2.90 | 2.69 |
| TTGGAAGAATTGTCTTG | UCRC | 24.44 | 8.32 | 6.15 | -2.94 | 2.43 |
| GTGAAGGCTGGACAAGG | NKIRAS2 | 30.92 | 10.52 | 23.83 | -2.94 | 2.31 |
| CAGATTAAGTGCAGTGT | TSC22D2 | 23.19 | 7.78 | 13.28 | -2.98 | 2.10 |
| CTATGGTGTTCTTCAAG | LCN2 | 31.68 | 10.47 | 57.78 | -3.03 | 2.46 |
| GTTAAAAAAAAAAAAAA | CENTA1 | 26.28 | 8.55 | 22.38 | -3.07 | 2.35 |
| CAGAATAATATTTTTAA | HTATSF1 | 29.24 | 9.50 | 29.08 | -3.08 | 2.64 |
| GTATCTATGCAGTGGGA | FLJ21511 | 71.30 | 22.89 | 31.15 | -3.11 | 1.98 |
| ACTGCAGAGCGGTAGAG | C14orf65 | 38.66 | 11.99 | 22.73 | -3.22 | 2.01 |
| GGAGATGGAGCCACATT | PPP1R7 | 42.96 | 13.31 | 25.09 | -3.23 | 2.49 |
| AGGTATATATCTTTGCC | SH3D19 | 36.34 | 11.22 | 24.08 | -3.24 | 2.37 |
| GAATTCAGCACACTTTT | SAMD9 | 81.60 | 24.54 | 48.96 | -3.33 | 2.48 |
| GTAGAGCTTGACCCAAG | PARP6 | 28.04 | 8.42 | 23.08 | -3.33 | 2.41 |
| CTTTTCTTCAAGGAAAG | C3orf9 | 49.49 | 14.67 | 18.00 | -3.37 | 2.65 |
| GTAAATTTGCAGCCATT | SUDS3 | 26.29 | 7.79 | 21.61 | -3.37 | 2.69 |
| GTCTTTCTGGGCCTTAC | UACA | 61.81 | 18.15 | 18.21 | -3.41 | 2.33 |
| TGTAAAGCAAAATTGGA | SPINK5 | 75.54 | 21.75 | 21.90 | -3.47 | 1.99 |
| CTGAGCTGGGACTGCAG | KRT17 | 25.18 | 7.23 | 4.74 | -3.48 | 2.27 |
| GGGTGGGGGCTGTCAGG | BAT1 | 26.19 | 7.36 | 7.22 | -3.56 | 2.32 |
| TTCTAGAGAGTAGGACT | CKAP2 | 20.40 | 5.73 | 8.12 | -3.56 | 2.41 |
| ATGAGGCAGCTGAGGTG |  | 29.32 | 8.19 | 23.09 | -3.58 | 2.77 |
| TTTTAATCTTGTTGGGA | KIAA1128 | 20.30 | 5.45 | 0.00 | -3.73 | 2.00 |
| CTGCGGAAGATGGGATC | CTNNBIP1 | 95.03 | 25.29 | 32.74 | -3.76 | 1.98 |
| CTTCCTGTATAGCCTGC | FLJ22671 | 158.67 | 39.99 | 85.74 | -3.97 | 2.24 |
| AACTATAGAAAAAAATC | SEC24B | 27.91 | 6.89 | 11.32 | -4.05 | 2.11 |
| AAAGTCATTGGTAGGCC | GATAD1 | 23.81 | 5.81 | 15.24 | -4.10 | 2.27 |
| GGGTCTGAGGGGAGAGA | SLURP1 | 494.90 | 120.67 | 155.42 | -4.10 | 2.06 |
| AAAATAAACTTGAATAA | PTEN | 20.42 | 4.95 | 5.89 | -4.13 | 2.45 |
| TGGAGCACCAGCCTCGG | OSBPL3 | 23.89 | 5.73 | 12.47 | -4.17 | 2.47 |
| GAGGGGCACTGGGGAAG | NUDT18 | 38.67 | 9.19 | 14.62 | -4.21 | 2.41 |
| AGCCACTGCGCCCAGCC | KIAA1840 | 24.42 | 5.71 | 23.70 | -4.28 | 2.15 |
| GAGGAGATGGCCAGGGC | DKFZp434N035 | 20.37 | 4.71 | 20.26 | -4.32 | 2.69 |
| CAGTCTTTTGCAGTACC | LOC161527 | 22.67 | 5.11 | 8.15 | -4.44 | 2.87 |
| AATGTGTTACTGTATAC | SLK | 30.39 | 6.66 | 12.72 | -4.57 | 2.84 |
| TGAAACAAAATGAAAAA | TMEM159 | 28.57 | 6.16 | 12.65 | -4.64 | 2.09 |
| GCCGCCCTCAGACCCCT | RPL32 | 26.74 | 5.73 | 9.38 | -4.67 | 2.10 |
| TCCACCAAGTCTGAGCC | SPINK5 | 2487.65 | 489.40 | 472.13 | -5.08 | 2.27 |
| ATTTCCATTAAAGTCTG | HR | 25.68 | 4.91 | 4.45 | -5.23 | 2.38 |
| AGACTCCATCAGGCAGT | CENPL | 21.40 | 3.93 | 10.06 | -5.44 | 2.41 |
| AGGCAGAGGTTGCAGTG | TRIO | 21.99 | 4.03 | 13.39 | -5.45 | 2.27 |
| TGATAATGGTACTATGG | ACOX2 | 22.65 | 4.09 | 22.79 | -5.53 | 2.69 |
| GGGGACAGAACAAAGAA | KIAA1794 | 20.96 | 3.76 | 10.96 | -5.57 | 2.63 |
| TGTAGGCTGCATAATTT | HEPHL1 | 54.67 | 9.34 | 5.97 | -5.85 | 2.06 |
| TGGAACTGTGAGTCAAT | SIGLEC8 | 21.42 | 3.63 | 7.61 | -5.90 | 2.71 |
| ACGCGAAGACGGATTTC | DHX32 | 23.29 | 3.41 | 6.87 | -6.82 | 1.96 |
| CCCTGTTCAGCTACTCC | TIE1 | 21.08 | 3.08 | 9.49 | -6.85 | 2.42 |
| CCCTTGAGGAGCTGGCC | SPRR1A | 35.07 | 5.09 | 4.89 | -6.89 | 2.16 |
| TCGTTTCCTTCCTGCTC | DHCR7 | 20.79 | 2.56 | 13.08 | -8.11 | 2.21 |
| AAGGCCATCTCTGTTTC | HOP | 23.89 | 2.83 | 5.52 | -8.44 | 2.40 |
| TTATTATTAAATTTTCT | SCEL | 268.13 | 30.98 | 38.59 | -8.66 | 2.26 |
| ACATTTTAGAAGTGGAG | ATP5S | 29.76 | 3.41 | 13.98 | -8.72 | 3.07 |
| CCAGAACAGACTGGGTG | RPL30 | 23.12 | 2.00 | 11.65 | -11.57 | 2.12 |
| TGTCAGAGCCATTTGGT | CIDEA | 23.75 | 1.28 | 4.44 | -18.53 | 2.00 |
| AAGTTGAGGCAGAGATG | HLA-A | 0.00 | 28.29 | 5.94 | -Normal | 1.98 |
| TGAGTGACAGAAATGGT | PIGR | 0.00 | 23.21 | 23.94 | -Normal | 2.19 |
