# Supplementary material for: Up regulation in gene expression of chromatin remodelling factors in cervical intraepithelial neoplasia
Source: BMC Genomics. 2008 Feb 4;9:64. doi: 10.1186/1471-2164-9-64 (PMC2277413; doi:10.1186/1471-2164-9-64)
Supplement: Additional file 2 — Genes differentially expressed in the later stages of neoplasia. Scales tags differentially expressed between CIN I/II and CIN III. [file 1471-2164-9-64-S2.doc]

**Supplemental Table 2**

Tags differentially expressed between moderate to severe dysplasia.

| Tag | Symbol | NC_Mean | CINIAndII_Mean | CINIII_Mean | Fold Change | PS_CINIAndII vs CINIII |
| --- | --- | --- | --- | --- | --- | --- |
| ACAATCATTTTCATCCC | CP | 2.36 | 51.19 | 4.63 | -11.06 | 1.98 |
| TTTCCCATAAAAAAAAA | CAPN13 | 19.68 | 27.82 | 3.41 | -8.17 | 2.18 |
| GAGGCGCTGCTGCCACC | TSPYL2 | 6.56 | 24.63 | 3.34 | -7.36 | 2.06 |
| TCATCTGGAATAATACT | CHFR | 4.15 | 22.35 | 3.23 | -6.93 | 2.87 |
| TAGGTCAGGACCTTGGC | PTP4A3 | 5.50 | 22.97 | 3.56 | -6.46 | 2.49 |
| ATAATGATAATAAAGGA |  | 22.68 | 20.63 | 3.55 | -5.80 | 2.09 |
| CTGGGTGCCCGGGAAGT | IGHG1 | 14.38 | 123.66 | 23.04 | -5.37 | 2.03 |
| TAATTTTTCTAAGGTGC | LOC653790 | 35.54 | 57.65 | 10.98 | -5.25 | 2.93 |
| CAATACTTATAAGACTT | CYP24A1 | 20.71 | 48.10 | 9.62 | -5.00 | 2.03 |
| AGGAGGTATCAGTCTAG | GNLY | 10.76 | 21.50 | 4.59 | -4.68 | 3.22 |
| GTTCATAGGTCATAGAA | EXOC4 | 7.18 | 22.60 | 4.96 | -4.56 | 3.13 |
| CAGCCAAATAAAAGGTG | FBXO44 | 8.98 | 20.22 | 4.45 | -4.54 | 2.19 |
| TAGAAGGTGGAAGAAGG | C1orf54 | 16.09 | 38.95 | 8.90 | -4.38 | 1.99 |
| GTCCAACAACACTCAAA | ATP5L | 7.72 | 20.16 | 4.63 | -4.35 | 2.16 |
| CTTTTTTCCTATGGTTA | KGFLP1 | 13.31 | 20.40 | 4.78 | -4.27 | 2.26 |
| AGCAGAGCCCAGGATGG | C9orf37 | 20.26 | 24.49 | 5.76 | -4.25 | 2.09 |
| GCATTTCAGGGTATAAG | SNX4 | 10.08 | 24.13 | 5.74 | -4.20 | 2.61 |
| GCACCAAAGCCACCAGA | CCL3L1 | 7.28 | 23.59 | 5.67 | -4.16 | 2.04 |
| TACAAACCTGGATTTTT | MT1F | 5.50 | 22.70 | 5.49 | -4.13 | 2.07 |
| GGGCCAACCCCACTAAA |  | 27.28 | 26.94 | 6.93 | -3.89 | 2.53 |
| ACAACTCCTGCAGTTTT | ATXN7 | 5.98 | 20.07 | 5.53 | -3.63 | 1.99 |
| ATGGTACTAAATGTTTT | SPIRE1 | 11.45 | 33.18 | 9.26 | -3.58 | 2.44 |
| CTGAAGGCTGAAAGACA | PECAM1 | 28.28 | 72.72 | 20.50 | -3.55 | 2.14 |
| AGTTTCAGAGAGTAATG |  | 14.40 | 20.39 | 5.82 | -3.51 | 2.25 |
| CCCAAACTTTGAGAATT | KCTD12 | 24.03 | 31.63 | 9.04 | -3.50 | 3.05 |
| GCAATGAAAATTTTAAG | APRIN | 12.49 | 22.69 | 6.68 | -3.39 | 2.76 |
| TCAGAACAGTCCAGACT | GRSF1 | 3.62 | 26.04 | 8.00 | -3.25 | 2.99 |
| TAAAATGTTTATAAATT | LOC401504 | 13.74 | 34.51 | 11.13 | -3.10 | 2.05 |
| CCACTGCACTCCAGACT | CHMP4C | 23.51 | 24.79 | 8.12 | -3.05 | 2.46 |
| GTGGCAGCGCTCATAGC | STMN3 | 16.94 | 27.97 | 9.37 | -2.98 | 2.28 |
| AATTAAAGTATATAGCG | FBXO9 | 18.53 | 27.25 | 9.19 | -2.96 | 1.98 |
| TGGTATTTCGGATCAGC | ALDH16A1 | 11.53 | 26.77 | 9.26 | -2.89 | 2.28 |
| CACTATGTAAACAGACA | WASPIP | 14.68 | 23.58 | 8.18 | -2.88 | 2.18 |
| TGACTGGTCACTCCTGG | DMWD | 13.71 | 25.99 | 9.11 | -2.85 | 2.06 |
| CGCGTGCACACGGGCCT | ZBTB7A | 3.57 | 23.83 | 8.55 | -2.79 | 2.06 |
| TGGACAAGCTAAGTGGG | BASP1 | 18.68 | 27.55 | 9.93 | -2.77 | 2.30 |
| TTCTGTGCTGGACACCC | C1R | 38.98 | 59.62 | 21.54 | -2.77 | 2.61 |
| GTTGACTTTTTCAAGCA | APITD1 | 9.02 | 24.67 | 9.13 | -2.70 | 2.31 |
| AAACCCCAATAAATATC | IGL@ | 596.06 | 2503.40 | 953.64 | -2.63 | 2.01 |
| GGGGCTTCCAGACCCCG | PHF15 | 27.59 | 24.22 | 9.35 | -2.59 | 2.73 |
| AGTGCAGGGAGAAGGGC | IGL@ | 141.94 | 779.63 | 304.58 | -2.56 | 2.22 |
| TGTAAAGATTTTTACAA | CCNL1 | 25.12 | 40.49 | 16.05 | -2.52 | 2.23 |
| TACATTTTATTTGATAT | DUSP12 | 27.18 | 39.00 | 15.58 | -2.50 | 2.63 |
| GTGTGCTGGCTTAAAAT | IFNGR2 | 28.32 | 26.35 | 10.60 | -2.49 | 2.69 |
| GGAATAAAAGTCAGTGG | SRPRB | 19.79 | 46.07 | 19.18 | -2.40 | 2.20 |
| TGTTAATTTATTGAGTG | KIAA0194 | 24.47 | 33.47 | 14.08 | -2.38 | 2.17 |
| GGTGGTTCACATACACA | TPI1 | 20.90 | 23.66 | 10.27 | -2.30 | 2.12 |
| TGTTTGAATTCACAAGG | THSD1 | 21.60 | 20.41 | 8.87 | -2.30 | 1.96 |
| GACCACGAATATTCTTT | CTSH | 185.12 | 103.83 | 45.37 | -2.29 | 2.69 |
| TCCGTGGTTGGGTGCAC | BASP1 | 38.98 | 28.66 | 12.54 | -2.29 | 2.54 |
| TTTTAAAATAAATTTTA | TTYH3 | 26.17 | 40.70 | 18.46 | -2.21 | 2.00 |
| TAGCAATTGCACTGTGC |  | 25.81 | 51.47 | 24.00 | -2.14 | 2.37 |
| TATTGGCCTGGAGGTGG | CTSD | 21.49 | 59.04 | 27.64 | -2.14 | 2.01 |
| AGGTCAAGAGATCGAGA |  | 37.92 | 33.30 | 16.42 | -2.03 | 1.99 |
| GCAAATGTACAGATTTT | CLASP1 | 47.11 | 36.98 | 18.28 | -2.02 | 2.32 |
| GAAAGAATTTCCTGTAG | TMCO4 | 18.01 | 22.40 | 45.43 | 2.03 | 2.06 |
| TACGCTTGGTCCAAGAT | CYB5R1 | 7.28 | 12.43 | 25.25 | 2.03 | 2.52 |
| CTAAATATTCTTTCCTA | SMC3 | 11.39 | 15.56 | 31.94 | 2.05 | 2.18 |
| TTTCCAATCTCTCTCTC | VEGF | 13.23 | 10.60 | 21.83 | 2.06 | 2.27 |
| CCCCGCCAAGTACTGCA | WDR18 | 84.27 | 54.24 | 112.63 | 2.08 | 2.11 |
| CAATCATTCCCCTTTGC | ANXA11 | 28.85 | 23.75 | 49.98 | 2.10 | 2.19 |
| GGGAATAAACCAGCATT | MVD | 42.32 | 27.65 | 58.36 | 2.11 | 2.35 |
| CTGTTCATCTATGAAGT | GFM2 | 14.50 | 13.02 | 27.68 | 2.13 | 2.32 |
| TTTTATTTCAAGGTTCT | PANK2 | 18.37 | 10.17 | 21.64 | 2.13 | 2.04 |
| GGGCCTGTGCCCACCCC | SLC16A3 | 72.23 | 71.45 | 153.36 | 2.15 | 1.97 |
| GCTTATGCTTCTGGTCC | MRPL17 | 39.00 | 27.40 | 60.14 | 2.19 | 2.08 |
| GGCCAGGTGGGGCTGCC | PCOLN3 | 33.99 | 26.94 | 59.19 | 2.20 | 2.10 |
| AAAAAGAAAAAAAAAAA | DUSP26 | 13.18 | 12.54 | 27.70 | 2.21 | 2.00 |
| CTTGTAATCCCAGCACT | PLCB1 | 25.71 | 16.30 | 36.01 | 2.21 | 2.08 |
| GAGAATTAATCCCACCT | GPIAP1 | 26.29 | 24.53 | 54.27 | 2.21 | 2.04 |
| AACCCGGGAGGAGGAGG | IL10RB | 34.74 | 24.95 | 55.87 | 2.24 | 2.23 |
| GTGAAGGCTGGACAAGG | NKIRAS2 | 30.92 | 10.52 | 23.83 | 2.27 | 2.06 |
| AGGTCAGGAGTTTGAGA | KIAA0226 | 13.23 | 9.87 | 22.57 | 2.29 | 2.34 |
| TTCTGTGTTTCAGTAAA | MUC20 | 107.13 | 75.82 | 173.44 | 2.29 | 2.11 |
| AGCAGAGCGATGGAGCA | DTYMK | 39.32 | 26.58 | 61.43 | 2.31 | 2.03 |
| CCTATAATCCCAGTGCT | VPS24 | 20.31 | 18.65 | 43.53 | 2.33 | 2.25 |
| TGCCTCGTGAATTTGCT | LOC113444 | 29.84 | 14.84 | 34.74 | 2.34 | 2.24 |
| CTATTCCATTTTGCAGC | IKBKG | 17.34 | 18.44 | 43.18 | 2.34 | 2.77 |
| GACCAGCTGCCCGGACC | GDPD3 | 38.16 | 25.01 | 58.94 | 2.36 | 2.02 |
| TCATACCATTGGTTTTC | RICS | 22.34 | 13.02 | 31.26 | 2.40 | 2.64 |
| GGCAACAGAGCAAGACT | ZNF587 | 20.22 | 10.25 | 25.05 | 2.44 | 2.72 |
| TCTTGCAAGTCTATGTT | RPS24 | 16.26 | 10.46 | 25.94 | 2.48 | 1.99 |
| TTCCAGCCAATGGATGA | TMEM106C | 10.71 | 16.40 | 41.03 | 2.50 | 2.21 |
| GTACAAAAATAAGTGGT | RAB1A | 19.82 | 12.87 | 32.42 | 2.52 | 2.70 |
| CAGGGGTTGGGGTTGGG | ATG16L2 | 35.20 | 11.80 | 30.47 | 2.58 | 2.08 |
| GAACCTTGAGAACCCAG | FOXK2 | 16.16 | 12.54 | 32.48 | 2.59 | 2.10 |
| ACCGGGAGCCGCGGTCT | SERPINB3 | 22.12 | 8.32 | 21.65 | 2.60 | 2.61 |
| GTGGGACCATTTCAAGA | FRMD5 | 13.66 | 13.07 | 34.02 | 2.60 | 2.25 |
| CTTTTGGCTGCCCTGAG | FDFT1 | 17.39 | 13.01 | 34.17 | 2.63 | 2.60 |
| ACACAGCAAGACGAGAA | RPL34 | 547.39 | 277.53 | 742.92 | 2.68 | 1.96 |
| TATATAACAGAATCAAG | TPMT | 10.10 | 9.28 | 25.02 | 2.69 | 2.33 |
| TCTTTGATCTGGTTTTA | SMARCC1 | 1.83 | 7.49 | 20.21 | 2.70 | 2.29 |
| GCGATGGCCGTGGAGAG | MMAB | 56.91 | 22.44 | 60.62 | 2.70 | 2.41 |
| TTTTGTTTTCTTGGGAA | GCNT2 | 2.36 | 7.44 | 20.37 | 2.74 | 2.72 |
| CAGCTGTAGTTGTTCAC | KIAA0174 | 27.10 | 16.43 | 45.03 | 2.74 | 2.67 |
| CCTGCCCCTTCCCCTGT | SNRPA | 14.42 | 17.71 | 48.90 | 2.76 | 2.91 |
| AGCCAAAAAAAAAAAAC | PCBP2 | 4.72 | 7.37 | 20.44 | 2.77 | 2.07 |
| TTAACCCTCTAGCTGAT | H3F3B | 42.59 | 17.23 | 48.15 | 2.79 | 2.39 |
| TCTGGGAGAGAACTTTT |  | 27.31 | 13.11 | 37.07 | 2.83 | 2.61 |
| TGTTTGTACATTTTTGT | B4GALT5 | 31.18 | 38.08 | 109.61 | 2.88 | 2.61 |
| CCATTGCACTTCAGCCT | LUZP2 | 12.46 | 6.96 | 20.12 | 2.89 | 2.00 |
| GAATTCCAGTTATCTGG | SLFN11 | 10.18 | 7.89 | 22.84 | 2.89 | 2.76 |
| GCATAATAGGTGTTAAG | LOC653665 | 5.50 | 7.14 | 21.43 | 3.00 | 1.99 |
| GTGGGGGGGAGGGAGAA | AHDC1 | 16.11 | 7.34 | 22.09 | 3.01 | 2.21 |
| CAGAATAATATTTTTAA | HTATSF1 | 29.24 | 9.50 | 29.08 | 3.06 | 2.61 |
| ATGGATGCACTGACTGA | NCLN | 11.43 | 6.81 | 20.84 | 3.06 | 2.81 |
| AATAAAGGTGCCTGAAA | HSBP1 | 30.96 | 13.78 | 42.34 | 3.07 | 2.97 |
| GTACTCTACTTCGTGTG | TUG1 | 9.04 | 9.09 | 28.05 | 3.09 | 2.63 |
| AAAGTGGAAACATTGGT | ATG5 | 5.94 | 7.01 | 21.71 | 3.10 | 2.08 |
| CTCCCTCCTCTCCTACC | TK1 | 10.70 | 7.49 | 23.88 | 3.19 | 2.53 |
| CCCGCCTCTTCACGGGC |  | 86.40 | 55.34 | 187.50 | 3.39 | 2.09 |
| GTGAAACCCCATCTCCA | C9orf139 | 16.63 | 8.36 | 29.43 | 3.52 | 2.92 |
| TCGTAATAGTTGATGTA | MIF | 17.24 | 7.32 | 26.27 | 3.59 | 2.59 |
| AGCCACCACGCCCGGCC | LOC441220 | 3.61 | 5.64 | 20.54 | 3.64 | 2.83 |
| GCGATGGGGGAGGGCGA | ZNF205 | 19.19 | 6.24 | 22.93 | 3.67 | 2.57 |
| ATGTAGAGTGTGGTTAT | TYMS | 7.77 | 8.63 | 32.33 | 3.75 | 2.10 |
| CCACTGCACTGGGCAAC | GALM | 10.17 | 5.64 | 21.23 | 3.76 | 2.45 |
| TAAAACGTGAAAAAAAA | PER3 | 11.39 | 6.13 | 24.29 | 3.96 | 2.23 |
| GTGAAACCCTGCCTCTA | MLLT6 | 23.85 | 6.63 | 27.27 | 4.12 | 2.53 |
| TTACTGCCTAGGGCGGT | TMEM25 | 1.78 | 5.04 | 20.85 | 4.13 | 1.99 |
| TGTATGACTCGTAGTCC | EEF1G | 1.78 | 4.79 | 20.03 | 4.18 | 2.36 |
| GCCCCTGCCTTAGGCCT | GSTM4 | 30.53 | 6.79 | 32.03 | 4.72 | 2.09 |
| CACTCCCCACTCTGAAA | B3GNT8 | 10.17 | 8.62 | 42.27 | 4.90 | 2.46 |
| GTTTTCCATAGTTGCCT | ATP10B | 26.26 | 10.97 | 57.38 | 5.23 | 2.14 |
| TGTCACACACAGACCCA | MRFAP1 | 1.83 | 3.64 | 23.67 | 6.50 | 2.44 |
| AAGAATCTGAAAAAAAA | NDUFB1 | 50.39 | 14.77 | 102.48 | 6.94 | 2.01 |
| ACAACTTTTATTGCCCT | BCAP31 | 10.67 | 3.51 | 26.56 | 7.57 | 2.43 |
| TGGGATGCGCTGAACCA | B3GNT3 | 10.14 | 3.38 | 37.09 | 10.97 | 2.43 |
