# Supplementary material for: Up regulation in gene expression of chromatin remodelling factors in cervical intraepithelial neoplasia
Source: BMC Genomics. 2008 Feb 4;9:64. doi: 10.1186/1471-2164-9-64 (PMC2277413; doi:10.1186/1471-2164-9-64)
Supplement: Additional file 3 — Tags mapping to the HPV 16 genome. Tags and scaled tag counts described. [file 1471-2164-9-64-S3.doc]

Supplemental Table 3. Tags* mapping to the Human Papillomavirus 16 genome.

| Tags | C1 | C2 | C3 | C4 | C5 | C6 |
| --- | --- | --- | --- | --- | --- | --- |
| CATGCGCCTAGAATGTGCTAT |  |  |  |  | 1.47 | 17.00 |
| CATGGCATTGGACAGGACATA |  |  | 48.00 |  | 6.00 | 13.00 |
| CATGCACGCTTTTTAATTACA |  |  | 236.00 |  | 47.00 | 12.00 |
| CATGGGGAGGAATATGATTTA |  |  | 6.00 |  |  | 6.00 |
| CATGCAACATAAATAAACTTA | 1.18 |  | 154.00 |  | 10.00 | 5.00 |
| CATGACACAATAGTTACACAA |  |  | 25.00 |  | 4.00 | 5.00 |
| CATGTCGTAGGTACTCCTTAA |  |  | 1.47 |  |  | 6.00 |
| CATGGGGATCCTTTGCCCCAG |  |  | 1.47 |  |  | 7.00 |
| CATGATAATATATGTTTGTGC |  |  | 1.47 |  |  | 8.00 |
| CATGTGTATGTATTAAAAATA |  |  | 31.00 |  | 3.00 | 12.00 |
| CATGTAGACGACACTGCAGTA |  |  | 4.00 |  |  | 13.00 |
| CATGGTAGATTATGGTTTCTG |  |  | 1.47 |  |  | 21.00 |

*scaled tag counts to tags per million.
