# Supplementary material for: Up regulation in gene expression of chromatin remodelling factors in cervical intraepithelial neoplasia
Source: BMC Genomics. 2008 Feb 4;9:64. doi: 10.1186/1471-2164-9-64 (PMC2277413; doi:10.1186/1471-2164-9-64)
Supplement: Additional file 4 — Genes concurrently expressed in all 16 libraries. Scales tags expressed at similar levels in all 16 libraries. [file 1471-2164-9-64-S4.doc]

**Supplemental Table 4**

Tags concordantly expressed between all libraries (P Score <0.45).

| Symbol | PS_CINIAndIIvsCINIII | PS_NVsCINIAndII | PS_NVsCINIII |
| --- | --- | --- | --- |
| NAT13 | 0.39 | 0.44 | 0.02 |
| NCF1 | 0.00 | 0.26 | 0.02 |
| IGHG1 | 0.35 | 0.38 | 0.03 |
| PSMB9 | 0.45 | 0.44 | 0.04 |
| SAMD4B | 0.33 | 0.40 | 0.05 |
| TTC1 | 0.43 | 0.12 | 0.08 |
| SSBP4 | 0.21 | 0.41 | 0.13 |
| LOC124446 | 0.39 | 0.35 | 0.14 |
| CDC2L2 | 0.42 | 0.35 | 0.16 |
| PLCE1 | 0.43 | 0.09 | 0.17 |
| SPG21 | 0.31 | 0.28 | 0.20 |
| BICD2 | 0.44 | 0.36 | 0.21 |
| NDUFA4 | 0.12 | 0.42 | 0.22 |
| CHMP5 | 0.36 | 0.43 | 0.22 |
| H2AFZ | 0.37 | 0.10 | 0.24 |
| C1orf116 | 0.40 | 0.29 | 0.24 |
| LOC283340 | 0.30 | 0.16 | 0.24 |
| SLC25A6 | 0.36 | 0.41 | 0.26 |
| S100A16 | 0.45 | 0.20 | 0.27 |
| UNC93B1 | 0.11 | 0.29 | 0.29 |
| PECI | 0.18 | 0.04 | 0.32 |
| TINAGL1 | 0.35 | 0.26 | 0.32 |
| AMZ2 | 0.12 | 0.36 | 0.32 |
| MRP63 | 0.20 | 0.45 | 0.32 |
| RPL13 | 0.44 | 0.42 | 0.35 |
| HEXB | 0.30 | 0.41 | 0.35 |
| SPINT2 | 0.42 | 0.33 | 0.35 |
| DRAP1 | 0.31 | 0.37 | 0.36 |
| STX5 | 0.45 | 0.43 | 0.36 |
| LAMP2 | 0.40 | 0.30 | 0.37 |
| NCKIPSD | 0.39 | 0.10 | 0.37 |
| SYNGR2 | 0.06 | 0.36 | 0.37 |
| ABHD3 | 0.02 | 0.25 | 0.38 |
| GNG10 | 0.32 | 0.32 | 0.38 |
| RPS2 | 0.01 | 0.20 | 0.42 |
| FBF1 | 0.05 | 0.32 | 0.42 |
| KLHDC2 | 0.29 | 0.11 | 0.43 |
| MRPL45 | 0.06 | 0.05 | 0.44 |
| PDHA1 | 0.30 | 0.45 | 0.45 |
| PRF1 | 0.24 | 0.16 | 0.45 |
| ABCB10 | 0.04 | 0.10 | 0.45 |
