# Supplementary material for: Up regulation in gene expression of chromatin remodelling factors in cervical intraepithelial neoplasia
Source: BMC Genomics. 2008 Feb 4;9:64. doi: 10.1186/1471-2164-9-64 (PMC2277413; doi:10.1186/1471-2164-9-64)
Supplement: Additional file 5 — Genes differentially expressed between normal cervical epithelium and severe dysplasia. Scales tags differentially expressed between normal and CIN III. [file 1471-2164-9-64-S5.doc]

**Supplemental Table 5**

All tags differentially expressed in CIN III when compared to Normal Cervical Tissue

| **Tag** | **Symbol** | **NC Mean** | **CINIAndII Mean** | **CINIII Mean** | **Fold Change** | **PS_NC vs CINIII** |
| --- | --- | --- | --- | --- | --- | --- |
| TTACGAGGAAGAAACTA | SEC13L1 | 0.00 | 19.64 | 24.89 | - Normal | 2.07 |
| ATAGAGGCAATGCATTA | MORF4L2 | 1.78 | 18.65 | 35.20 | 19.78 | 3.36 |
| TGTCACACACAGACCCA | MRFAP1 | 1.83 | 3.64 | 23.67 | 12.90 | 2.14 |
| TGGCCGGGGAAGATGGA | WDR18 | 1.79 | 8.82 | 22.91 | 12.80 | 2.50 |
| TGTATGACTCGTAGTCC | EEF1G | 1.78 | 4.79 | 20.03 | 11.26 | 2.41 |
| ACACAGTATTCGCTCTT | GPR180 | 1.78 | 7.50 | 20.01 | 11.24 | 2.37 |
| TCTTTGATCTGGTTTTA | SMARCC1 | 1.83 | 7.49 | 20.21 | 11.02 | 2.78 |
| AAGTTGGTGCTAATAAA | AMZ2 | 1.79 | 21.96 | 16.24 | 9.07 | 2.34 |
| TTTTGTTTTCTTGGGAA | GCNT2 | 2.36 | 7.44 | 20.37 | 8.62 | 2.67 |
| GGAGTCATTGTCCACAT | PSMB3 | 7.28 | 33.65 | 51.33 | 7.05 | 2.50 |
| CTCCCTTTTACATTCTG | HOXB7 | 5.46 | 24.98 | 38.39 | 7.03 | 1.98 |
| AGAAATCACTGTTGCAA | HADHSC | 3.57 | 11.63 | 25.08 | 7.03 | 2.05 |
| ATCCGCCTGCTTTGGCC |  | 3.61 | 10.55 | 25.03 | 6.93 | 2.70 |
| AGAGAGATTATAAGAAG | FAM33A | 3.56 | 14.94 | 24.47 | 6.87 | 2.08 |
| GCTACTATTAGATCAGG | MCM4 | 8.95 | 30.88 | 60.00 | 6.70 | 2.33 |
| TAGCACAAAGTTTCTCT | PAM | 3.61 | 27.87 | 23.56 | 6.52 | 2.48 |
| TGGGAAAACTCCGCCTT | CYB561 | 3.62 | 26.42 | 21.56 | 5.95 | 2.29 |
| TCTGCATCTTGGACGCC | ALDH1L1 | 10.17 | 33.73 | 59.73 | 5.87 | 2.38 |
| GGAGAGACAGGTAGTTA | ZFP91 | 4.14 | 10.66 | 23.81 | 5.75 | 2.02 |
| AGCCACCACGCCCGGCC | LOC441220 | 3.61 | 5.64 | 20.54 | 5.68 | 2.76 |
| ATGTTAATAAAATAGGC | GPC4 | 4.72 | 36.50 | 25.92 | 5.49 | 2.23 |
| CATCTGTGAGCTTTAGA | DAP | 18.17 | 57.37 | 84.86 | 4.67 | 2.54 |
| TCAACAGCGTTCCTAGA | BID | 5.99 | 29.19 | 27.60 | 4.61 | 2.06 |
| GATAATGATTTTTTAAC | RAB27A | 5.34 | 16.22 | 24.07 | 4.51 | 1.97 |
| GTGATGTAAGATGTTGA | SRXN1 | 5.45 | 21.22 | 24.46 | 4.49 | 1.98 |
| GTGGCGGGTGCCTGTAG | SORBS2 | 3.61 | 20.83 | 16.03 | 4.44 | 1.97 |
| GTACATCCTTTGTCACT | ARFIP2 | 5.36 | 27.42 | 23.67 | 4.42 | 2.40 |
| GCTTGTTAAGATCAGGA | AHCYL1 | 7.82 | 22.81 | 33.76 | 4.32 | 2.11 |
| GGCCTCGGCCTCGGCGG | FRAT2 | 5.92 | 19.02 | 25.26 | 4.27 | 2.99 |
| CCCGCCCCCGCCTTCCC | TOMM40 | 5.41 | 24.42 | 23.09 | 4.26 | 2.05 |
| CTTTCCTTTTCTTCCTT | UCP2 | 13.84 | 58.80 | 58.70 | 4.24 | 2.10 |
| GGAATATGCAGAATTTC | CRLS1 | 20.43 | 97.91 | 84.44 | 4.13 | 2.59 |
| CTGTACTAGGTGCTGAA | RUSC1 | 12.60 | 32.76 | 50.18 | 3.98 | 2.10 |
| GGAAGTGCCACTGGGAT | F11R | 9.07 | 13.77 | 35.26 | 3.89 | 2.05 |
| TTCCAGCCAATGGATGA | TMEM106C | 10.71 | 16.40 | 41.03 | 3.83 | 2.38 |
| GTTAATTGCTAGTTGGT | SELS | 9.60 | 26.71 | 35.79 | 3.73 | 2.31 |
| GAGTGGAGAGTTTATTC | USP9X | 10.92 | 38.39 | 40.66 | 3.72 | 2.60 |
| TCATTTATAAATTTTCT | BDP1 | 6.56 | 25.90 | 24.03 | 3.66 | 2.53 |
| TTGTATTTCAAATCTCT |  | 9.70 | 23.37 | 35.35 | 3.64 | 2.75 |
| GAAATATTGCTTAATTT | HERC4 | 5.41 | 22.16 | 19.17 | 3.54 | 2.23 |
| TGTTTGTACATTTTTGT | B4GALT5 | 31.18 | 38.08 | 109.61 | 3.52 | 2.24 |
| TACGCTTGGTCCAAGAT | CYB5R1 | 7.28 | 12.43 | 25.25 | 3.47 | 2.58 |
| GGCCCTGGTGTTTGCAC | GAK | 16.12 | 33.46 | 55.64 | 3.45 | 2.02 |
| AGCAGGCTCAGCCTAGG | S100P | 22.25 | 74.99 | 76.36 | 3.43 | 2.04 |
| CCTGCCCCTTCCCCTGT | SNRPA | 14.42 | 17.71 | 48.90 | 3.39 | 2.64 |
| GGGAGGAACAGACTGGC | POP4 | 7.70 | 19.36 | 25.89 | 3.36 | 2.04 |
| GCCTCTGCCAGATGGCT | BAP1 | 14.26 | 38.22 | 47.83 | 3.35 | 2.34 |
| GGCACCGTGCTGCTCCT | LOC440335 | 37.77 | 96.12 | 126.07 | 3.34 | 2.60 |
| GTTAAATGACTTCCCTT | C1orf186 | 19.15 | 57.47 | 63.89 | 3.34 | 2.01 |
| CCCCTAAGTATTCCTTT | NCOR1 | 12.65 | 38.27 | 42.02 | 3.32 | 2.36 |
| GAGGCCAGTGAAAGCTG | RPN1 | 15.69 | 51.34 | 51.85 | 3.31 | 2.18 |
| GAGAGGGCAGACTGTGC | RNF26 | 10.17 | 20.20 | 33.59 | 3.30 | 2.48 |
| TTGAAAGGTTTCTATGG | UAP1 | 23.33 | 51.65 | 76.56 | 3.28 | 2.87 |
| ATGGCGCCTCCTGCTCG | RPL18 | 7.24 | 19.54 | 22.96 | 3.17 | 2.19 |
| TGGACAGTGCACGTGCC | EYA2 | 26.19 | 53.95 | 82.24 | 3.14 | 2.73 |
| TACCTCTGATTAATAAA | S100P | 125.67 | 265.94 | 391.31 | 3.11 | 2.13 |
| GCCTTTCTAATAAACTG | RPS6KA1 | 9.65 | 35.01 | 29.64 | 3.07 | 2.01 |
| CACCCCTGATGTTCGCC | CKB | 55.26 | 208.36 | 165.75 | 3.00 | 2.14 |
| GAAAAAGATGTGTCAGC | GLT8D1 | 9.56 | 27.72 | 28.40 | 2.97 | 2.17 |
| CCGCTTCTGCCGGTGCC | NOP17 | 17.98 | 42.95 | 52.32 | 2.91 | 2.08 |
| TATAGTGGCTAACTTAA | PIM3 | 7.19 | 21.99 | 20.48 | 2.85 | 2.38 |
| TGAATTTATTACTCCCG | IMMP1L | 7.29 | 14.75 | 20.51 | 2.81 | 2.27 |
| CTAAATATTCTTTCCTA | SMC3 | 11.39 | 15.56 | 31.94 | 2.80 | 3.17 |
| TATTATTAAAGAGGATT | RAB20 | 23.97 | 66.99 | 66.20 | 2.76 | 2.42 |
| TCCCTGGCAGAGGGCTT | CRIP2 | 63.13 | 146.23 | 173.31 | 2.75 | 3.13 |
| GGCCCTCTGAGCAACTG | PIN1 | 13.11 | 23.20 | 35.90 | 2.74 | 1.98 |
| AAGGTAACTAACGGAAG | DHFR | 10.07 | 28.04 | 27.47 | 2.73 | 2.68 |
| GACAGTGACGCAAGGAC | ZNF593 | 16.26 | 60.11 | 44.10 | 2.71 | 2.06 |
| CCTCCAGCTACAAAACA | KRT8 | 92.25 | 171.18 | 247.88 | 2.69 | 2.04 |
| CCTGTAATCCCATCATC | NT5C2 | 9.03 | 21.31 | 24.07 | 2.67 | 2.31 |
| ACTGACTATCAATCAAA | NEU1 | 13.27 | 27.94 | 35.24 | 2.66 | 1.97 |
| TGCAGTCCCTGTCCCCC | PRKDC | 23.33 | 30.73 | 61.45 | 2.63 | 2.08 |
| CTGGGACTGACAGCCTG | LSM4 | 36.42 | 80.83 | 95.86 | 2.63 | 2.06 |
| GTAATCTTATAAATTCT | BCKDHB | 15.59 | 34.00 | 40.67 | 2.61 | 2.01 |
| GAGAGAAAATTTTGTCC | TMEM9 | 34.03 | 61.17 | 88.70 | 2.61 | 2.49 |
| TTTTTATTTAGTATTGG | NEO1 | 11.93 | 27.99 | 31.00 | 2.60 | 2.60 |
| ATGAGCTATGAACCACT | TMEM4 | 11.96 | 39.42 | 30.68 | 2.56 | 2.75 |
| TGGAGAGCAACTTGGGA | AHCYL1 | 30.57 | 67.03 | 78.40 | 2.56 | 2.15 |
| GACGCGGCGCGCCCGGA | CDC42EP5 | 45.93 | 53.18 | 117.65 | 2.56 | 2.02 |
| ACTGAGGTGCCTCCCAA | FIBP | 10.71 | 28.86 | 27.32 | 2.55 | 2.04 |
| GGAGGCAGGTGGGGCTT | B4GALT2 | 9.49 | 27.07 | 24.03 | 2.53 | 2.45 |
| TAAAGCCCAGTAGCAGG | ILF2 | 11.44 | 20.16 | 28.69 | 2.51 | 2.23 |
| TATAAATAAAGCTCCTC | THRAP4 | 38.67 | 66.91 | 96.71 | 2.50 | 2.04 |
| GACCACACCGGGTCTGG | NUDT8 | 24.49 | 39.03 | 60.97 | 2.49 | 2.45 |
| CTATTCCATTTTGCAGC | IKBKG | 17.34 | 18.44 | 43.18 | 2.49 | 2.11 |
| TTTCCCTTCTAGTTTTG | TRFP | 9.59 | 24.39 | 23.71 | 2.47 | 2.12 |
| GTGTGTGGTGCCCCCAG | PRNPIP | 28.26 | 46.34 | 69.41 | 2.46 | 1.98 |
| GAAACTGAAGTTCTGCT | NIT1 | 25.28 | 47.85 | 60.79 | 2.40 | 2.31 |
| ATTTTTAAATAACCTGT | SF3B1 | 15.53 | 24.87 | 37.03 | 2.38 | 2.31 |
| TGTCGCTGGGGAGGAAG | HIGD2A | 25.79 | 43.31 | 60.60 | 2.35 | 2.50 |
| CGATGGTCCCCCACACC | PHB2 | 26.54 | 49.45 | 60.78 | 2.29 | 2.22 |
| GGCTTAGGATGTGAATG | RASIP1 | 11.91 | 17.08 | 26.84 | 2.25 | 2.20 |
| TACAGTTCCCTTTTTAA | SYF2 | 12.02 | 35.27 | 27.08 | 2.25 | 2.09 |
| GAATTCCAGTTATCTGG | SLFN11 | 10.18 | 7.89 | 22.84 | 2.24 | 1.97 |
| GAAGGCATCCTGGAGGT | PSMC3 | 33.56 | 70.52 | 74.62 | 2.22 | 2.36 |
| GCGGGGTACCCTAGGGA | NOMO3 | 42.49 | 62.29 | 94.31 | 2.22 | 2.20 |
| AAACCAGGGCCCTCTTC | COMMD9 | 19.68 | 26.94 | 42.76 | 2.17 | 1.99 |
| AAACATCCTATCATCTG | EBNA1BP2 | 25.08 | 39.85 | 54.49 | 2.17 | 2.02 |
| GTCTGAGCTCCCTGCCC | C22orf16 | 42.44 | 78.32 | 92.21 | 2.17 | 2.14 |
| CACTTCAAGGGCAGCCT | LY6E | 133.95 | 298.00 | 287.38 | 2.15 | 2.62 |
| AAGCCAGCCCCTGCAGT | PRKCSH | 15.12 | 39.70 | 32.22 | 2.13 | 1.98 |
| GGTGTATATGGAGCCCT | STOML2 | 17.31 | 28.21 | 36.84 | 2.13 | 2.05 |
| GAAAAAATGTTGATGGT | TCEAL4 | 39.10 | 89.27 | 83.13 | 2.13 | 2.07 |
| AGCACTTTTGCTGGTTC | FEZ2 | 23.87 | 27.54 | 50.64 | 2.12 | 2.33 |
| CAGGAACGGGGGTCTCC | MAP2K2 | 38.82 | 58.75 | 81.84 | 2.11 | 2.30 |
| AAAAAGAAAAAAAAAAA | DUSP26 | 13.18 | 12.54 | 27.70 | 2.10 | 2.30 |
| GAGAATTAATCCCACCT | GPIAP1 | 26.29 | 24.53 | 54.27 | 2.06 | 1.96 |
| CTAATAAATGCTTCTTC | RAB3D | 106.87 | 226.95 | 214.95 | 2.01 | 2.00 |
| TGCTTGACAAGTTTGAG | MGC9850 | 141.15 | 81.37 | 70.53 | -2.00 | 2.09 |
| TTCAGGGCTTCTAGAAC | EIF5 | 61.71 | 32.45 | 30.82 | -2.00 | 2.09 |
| ATGAATTAGCATATGCC | IFRD1 | 56.07 | 45.88 | 27.72 | -2.02 | 2.06 |
| TTGCCCAGGCTGGTCTT | HIF3A | 71.08 | 37.32 | 34.97 | -2.03 | 2.54 |
| AAAGAGAAAAAAAAAAA | CNOT1 | 32.23 | 12.52 | 15.70 | -2.05 | 2.27 |
| AGAAATAAAGATTATTT | AMPD2 | 48.53 | 33.18 | 23.35 | -2.08 | 2.34 |
| TTATATTTTCTTTTAAG | DOCK9 | 143.54 | 75.46 | 68.68 | -2.09 | 2.04 |
| GACGAGCTTTTTTCTCA | STUB1 | 45.25 | 38.91 | 21.63 | -2.09 | 1.97 |
| TGAGTCTGGCTGGGCTG | KLF6 | 44.83 | 16.28 | 21.38 | -2.10 | 2.04 |
| TGGGAGCCCTGTCCTCA | FAM102A | 74.62 | 30.29 | 35.28 | -2.11 | 2.21 |
| TATGTTATGATATTGTT | ELAC1 | 29.33 | 22.50 | 13.86 | -2.12 | 2.15 |
| AAGGTAACTTGGGTTTT | SLC2A3P1 | 40.00 | 23.85 | 18.89 | -2.12 | 2.26 |
| ACATTTTAGAAGTGGAG | ATP5S | 29.76 | 3.41 | 13.98 | -2.13 | 2.20 |
| CTAACTTCGTTTGTGCG | ENAH | 26.91 | 22.50 | 12.62 | -2.13 | 2.08 |
| AAAGCACAAGTGACTAG | KRT5 | 2209.45 | 1259.80 | 1006.52 | -2.20 | 2.21 |
| TTATTTAATAAAGAGTT | ANKRD12 | 62.81 | 47.78 | 28.40 | -2.21 | 2.35 |
| GTGATGGGGCCAGATGT | C6orf1 | 43.54 | 30.16 | 19.62 | -2.22 | 2.58 |
| TCCAGAATCCTGCTTAA | KLHL21 | 52.46 | 40.75 | 23.61 | -2.22 | 2.45 |
| ACAAGTACTGTATTTTT | CDH5 | 69.78 | 31.42 | 31.33 | -2.23 | 2.28 |
| GAAGGTGGAGGACAGAA | LY6D | 28.08 | 16.05 | 12.53 | -2.24 | 2.22 |
| TCATTTTGTGATATAGG | SVIL | 99.03 | 71.10 | 43.65 | -2.27 | 1.97 |
| GTAGGAAAGCTGCTTGG | NPEPPS | 90.08 | 58.48 | 39.65 | -2.27 | 2.70 |
| CTTATGTATTATGGCAA | KIF15 | 21.05 | 20.61 | 9.20 | -2.29 | 1.98 |
| AGGTCAAGAGATCGAGA |  | 37.92 | 33.30 | 16.42 | -2.31 | 2.01 |
| GCAGAGAGGAACCGCCG | KRT17 | 198.91 | 149.81 | 84.20 | -2.36 | 2.17 |
| GCTGGATGCGGATATTT | C9orf3 | 80.11 | 31.36 | 33.91 | -2.36 | 2.51 |
| CTAGGATGCGGAAATCC | CDKN2B | 15.53 | 20.76 | 6.57 | -2.37 | 2.26 |
| CTGGCCGCAAGACTGTG | CENPT | 22.05 | 20.81 | 9.20 | -2.40 | 2.38 |
| CTGTGATGTGACTCCTG | C1orf21 | 98.49 | 46.28 | 41.06 | -2.40 | 2.32 |
| GATTGAACCTCATCAAT | SC4MOL | 36.40 | 13.00 | 14.90 | -2.44 | 2.28 |
| GGGAAGGGCGCAACGGG | HIST3H2A | 22.67 | 21.62 | 9.19 | -2.47 | 2.13 |
| ATGTAAAATCAGAGGTT | KRT5 | 136.03 | 76.90 | 54.99 | -2.47 | 2.12 |
| AGATGAGATGACCACCA | KLF6 | 33.38 | 16.55 | 13.44 | -2.48 | 2.84 |
| TACTGAATATAACTATT | CITED2 | 25.71 | 12.24 | 10.27 | -2.50 | 2.45 |
| AATCAATAAAACTGATT | GPD2 | 32.28 | 22.31 | 12.86 | -2.51 | 2.21 |
| TATATTTCCACATTTTT | SYF2 | 43.52 | 26.92 | 17.03 | -2.56 | 2.41 |
| GCAAATGTACAGATTTT | CLASP1 | 47.11 | 36.98 | 18.28 | -2.58 | 2.43 |
| TAAAATGTTGTTCAGTT | FAM43A | 176.10 | 77.28 | 67.85 | -2.60 | 1.96 |
| CCTGGCCTAAAATGGGT | HSPB8 | 70.07 | 33.65 | 26.86 | -2.61 | 2.05 |
| GAGAACCACCTACCCCT | MAF | 34.26 | 16.52 | 13.04 | -2.63 | 2.09 |
| CCCAAACTTTGAGAATT | KCTD12 | 24.03 | 31.63 | 9.04 | -2.66 | 2.25 |
| TGCAGAAGTAGCTTCTC | AP3M2 | 24.45 | 17.00 | 9.16 | -2.67 | 2.12 |
| CAGGCGTGCACACAGCC | RECQL5 | 24.97 | 10.86 | 9.34 | -2.67 | 1.97 |
| CCTAACTCTGTACCGTT | APBB3 | 25.12 | 10.13 | 9.19 | -2.73 | 1.96 |
| CTTTTCTTCAAGGAAAG | C3orf9 | 49.49 | 14.67 | 18.00 | -2.75 | 3.01 |
| TTTAATAAACGGAGATT | SYNGAP1 | 28.17 | 33.01 | 10.21 | -2.76 | 2.14 |
| AAAGCGGGGCTGGAGAA | KRT17 | 4610.32 | 2448.63 | 1659.96 | -2.78 | 2.07 |
| TCAAATCACATTGAAGC | EHD4 | 35.83 | 13.77 | 12.89 | -2.78 | 2.36 |
| CAGTCTTTTGCAGTACC | LOC161527 | 22.67 | 5.11 | 8.15 | -2.78 | 2.68 |
| TGGAACTGTGAGTCAAT | SIGLEC8 | 21.42 | 3.63 | 7.61 | -2.81 | 2.00 |
| TTTTAAAAAAAAAAAAA | IKBKAP | 28.70 | 28.29 | 10.08 | -2.85 | 2.74 |
| ACCACAAATAAATAAAG | TMEM49 | 29.77 | 21.39 | 10.45 | -2.85 | 2.28 |
| CCAGCGCCAACCAGTCA | LYPD3 | 391.17 | 220.67 | 135.13 | -2.89 | 2.39 |
| CCTCCTATTACTGAAGT | RIOK3 | 149.94 | 79.12 | 51.58 | -2.91 | 2.09 |
| GGGGCTTCCAGACCCCG | PHF15 | 27.59 | 24.22 | 9.35 | -2.95 | 2.05 |
| TACATATCTGTTTAGTT | SMC6 | 26.91 | 11.01 | 9.01 | -2.99 | 2.72 |
| TCTGTTGTTCAAGTAAA | LOC284454 | 26.88 | 12.51 | 8.98 | -2.99 | 3.01 |
| CTCTTCAGGGTCAGTTC | KLHDC8B | 29.17 | 16.29 | 9.74 | -3.00 | 2.20 |
| CTTAAGAAAAATGCACT | ERRFI1 | 20.96 | 20.01 | 6.93 | -3.03 | 2.07 |
| AGTAAGGTGGCTTTGAT | C4orf27 | 24.50 | 9.94 | 7.97 | -3.07 | 2.12 |
| TTGAATATTAAATTTTT | C22orf9 | 36.96 | 28.76 | 12.00 | -3.08 | 2.59 |
| TTACAGCACAATATTTC | LYPLA1 | 28.06 | 16.84 | 8.99 | -3.12 | 2.71 |
| CAGATGTAGAAGAGAGC | RPS6KA3 | 24.62 | 14.58 | 7.67 | -3.21 | 2.15 |
| TAAATAAAAGATGTTGA | ZBTB11 | 32.96 | 24.53 | 10.19 | -3.24 | 2.17 |
| GATGTGCACGATGGCAA | KRT17 | 276.87 | 190.07 | 81.81 | -3.38 | 2.27 |
| CCACAGGAGAATTCAGG | NGRN | 22.73 | 10.96 | 6.71 | -3.39 | 2.95 |
| GCTTTTCTGGTCTTCGG | SPRR3 | 610.41 | 233.52 | 180.05 | -3.39 | 2.06 |
| GTCTTTCTGGGCCTTAC | UACA | 61.81 | 18.15 | 18.21 | -3.39 | 2.29 |
| CGAATGTCCTTTTTAGT | KRT5 | 428.58 | 155.25 | 125.72 | -3.41 | 2.46 |
| TGTAAAGCAAAATTGGA | SPINK5 | 75.54 | 21.75 | 21.90 | -3.45 | 2.00 |
| AAAATAAACTTGAATAA | PTEN | 20.42 | 4.95 | 5.89 | -3.47 | 2.32 |
| TGGAAGGAGTTTGAGGG | C17orf39 | 26.31 | 14.70 | 7.58 | -3.47 | 1.99 |
| CAGAACCTCAACGACCG | KRT17 | 176.03 | 84.72 | 50.43 | -3.49 | 2.00 |
| GCTGTGCCTGGAAGAAC | PRSS3 | 66.17 | 24.46 | 18.92 | -3.50 | 2.60 |
| TACTATTAAAAAAAAAA | POLB | 31.54 | 10.55 | 8.98 | -3.51 | 1.96 |
| AGCAGAGCCCAGGATGG | C9orf37 | 20.26 | 24.49 | 5.76 | -3.52 | 2.21 |
| CTGGTTTGTTTGCTTGC | CREG1 | 32.99 | 16.70 | 9.26 | -3.56 | 2.51 |
| AGCCTACAGGTGATTGG | AIM1L | 47.02 | 19.50 | 13.01 | -3.61 | 2.45 |
| GGGTGGGGGCTGTCAGG | BAT1 | 26.19 | 7.36 | 7.22 | -3.63 | 2.30 |
| TGTTTTGATGACAGGCA | FLJ43339 | 30.91 | 13.30 | 8.45 | -3.66 | 2.06 |
| TAACTTTGCCATCAGTT | WNK1 | 38.15 | 14.20 | 10.20 | -3.74 | 2.81 |
| TATAAAAGTCAAGCCCT | ZNF117 | 25.17 | 20.06 | 6.71 | -3.75 | 2.42 |
| CGGTGTTCTCTTTAATC | MAL | 296.66 | 124.94 | 79.10 | -3.75 | 1.99 |
| CTCCTGGGCGCCCACGC | CALML3 | 414.33 | 143.68 | 109.23 | -3.79 | 2.55 |
| TTTGTATATAGATACAG | KIAA1729 | 25.58 | 19.53 | 6.72 | -3.81 | 2.07 |
| AAATTGTATGTATTAAA | TMOD3 | 21.43 | 12.17 | 5.56 | -3.86 | 2.08 |
| AAGGATGCGGTGATGGC | TAF11 | 47.07 | 26.20 | 12.02 | -3.92 | 2.09 |
| GTCCGAGTGCACTGAAC | TM4SF1 | 57.77 | 31.25 | 14.71 | -3.93 | 2.22 |
| TTCATTTTTTTGAAAGA |  | 22.13 | 5.63 | 5.61 | -3.95 | 1.99 |
| CAGGGTGGGTGGAGCAA | KCNH2 | 96.83 | 30.36 | 24.54 | -3.95 | 2.01 |
| CTCAGCTGGGACTGCAG | KRT17 | 204.82 | 73.47 | 51.78 | -3.96 | 2.69 |
| CAATCTTGCAGTGAAGA | TMPRSS11B | 162.66 | 48.01 | 41.08 | -3.96 | 1.97 |
| TTGGAAGAATTGTCTTG | UCRC | 24.44 | 8.32 | 6.15 | -3.98 | 2.30 |
| CCTCACTGACAGACCAG | SPINK7 | 3010.40 | 1420.61 | 746.42 | -4.03 | 1.97 |
| GACCACGAATATTCTTT | CTSH | 185.12 | 103.83 | 45.37 | -4.08 | 2.89 |
| TGTAGGTCATTTTCAAG | GPR87 | 99.93 | 52.33 | 24.48 | -4.08 | 2.42 |
| TATTTTATTTGTGTATC | P2RY5 | 109.08 | 51.34 | 26.00 | -4.20 | 3.15 |
| GTGAATGTATGTTTCTG | EPB41L3 | 82.29 | 37.38 | 19.60 | -4.20 | 2.94 |
| GTTTCATCTCCAGGGAG | CRYAB | 171.57 | 97.74 | 39.70 | -4.32 | 2.59 |
| AAGGCCATCTCTGTTTC | HOP | 23.89 | 2.83 | 5.52 | -4.33 | 2.10 |
| CCATTGCACTCCAGCTT | CASC4 | 24.44 | 10.65 | 5.61 | -4.36 | 2.22 |
| AAGCTATAGCTTGCTGA |  | 28.12 | 12.36 | 6.42 | -4.38 | 2.60 |
| ATGCTTTTAATAAAAAC | NOXA1 | 56.05 | 27.35 | 11.71 | -4.79 | 2.28 |
| GAGCCTCAGGTGCTCCC | FNDC4 | 39.22 | 26.48 | 7.91 | -4.96 | 2.43 |
| TTTATTTATATAGATTT | USP15 | 22.11 | 7.87 | 4.38 | -5.04 | 2.41 |
| ATTCCTACATAGAGGCT | RNF39 | 24.44 | 9.77 | 4.81 | -5.08 | 2.31 |
| CTACCCAACAGTAGACA | CSNK1E | 23.74 | 7.71 | 4.56 | -5.20 | 2.09 |
| TCCACCAAGTCTGAGCC | SPINK5 | 2487.65 | 489.40 | 472.13 | -5.27 | 2.39 |
| CTGAGCTGGGACTGCAG | KRT17 | 25.18 | 7.23 | 4.74 | -5.31 | 2.61 |
| ATTTTGTAACCCAGATT | RBL2 | 31.05 | 15.54 | 5.70 | -5.45 | 2.43 |
| TTTCCTTTGCTGTGGTG | BZRAP1 | 25.56 | 23.37 | 4.56 | -5.60 | 2.70 |
| TTCCCTTACCCCTGTAC | LCE3D | 2050.19 | 1155.76 | 362.72 | -5.65 | 2.00 |
| ATGACAGATGGTGTGAA | HOP | 255.47 | 109.09 | 44.77 | -5.71 | 2.46 |
| TTAAAGTTTTATTAAGT | RBMX | 20.25 | 12.45 | 3.52 | -5.75 | 2.57 |
| ATTTCCATTAAAGTCTG | HR | 25.68 | 4.91 | 4.45 | -5.77 | 2.15 |
| ATAGCTGTAAGTCAGGC | JMY | 44.63 | 25.35 | 7.68 | -5.81 | 2.16 |
| ATAATGATAATAAAGGA |  | 22.68 | 20.63 | 3.55 | -6.38 | 2.44 |
| AGTCCTGCTTCTAGCTC | CDKN2B | 23.95 | 9.32 | 3.67 | -6.53 | 2.56 |
| TTATTATTAAATTTTCT | SCEL | 268.13 | 30.98 | 38.59 | -6.95 | 2.17 |
| CCCTTGAGGAGCTGGCC | SPRR1A | 35.07 | 5.09 | 4.89 | -7.17 | 2.05 |
| CAAGGATAAGAGGTTCT | TBXAS1 | 26.54 | 24.04 | 3.56 | -7.46 | 2.71 |
| ATCCAGAGGCTGAGATC | KRT5 | 54.74 | 33.80 | 7.01 | -7.81 | 2.59 |
| ATTTGTTGATTTGGGGG | LOC441212 | 26.32 | 18.25 | 3.34 | -7.88 | 2.97 |
| GCAATAAGTGTACTATG | SPINK5 | 130.91 | 28.97 | 15.53 | -8.43 | 2.12 |
| TAACTGGCCTTACGATG | DSG1 | 39.18 | 19.02 | 4.59 | -8.53 | 2.07 |
| TGTAGGCTGCATAATTT | HEPHL1 | 54.67 | 9.34 | 5.97 | -9.16 | 2.21 |
| TCTAAGCTTGTTCCAGC | FAM79B | 63.05 | 19.16 | 6.86 | -9.19 | 2.19 |
| TCCGAAACCTACGATTT | RAB4A | 23.17 | 4.56 | 2.30 | -10.09 | 2.29 |
| ATTGCGCCACTGCACCC | FLJ16165 | 25.52 | 19.02 | 2.30 | -11.11 | 2.39 |
| TATTATCTGATAAGAAT | CLDN17 | 42.15 | 19.37 | 3.34 | -12.62 | 2.05 |
| GATGGAATAAACTTGTG | PPP1R14C | 29.05 | 5.11 | 2.30 | -12.63 | 2.01 |
| GAGTGAGATCTTTTGAG | FAM43A | 20.78 | 4.03 | 1.08 | -19.31 | 2.35 |
| AGTGAGGCGGGCGAGGC | CSDA | 20.87 | 6.64 | 1.08 | -19.39 | 3.11 |
| AGGAAATGAAAGTGGCT | EDN3 | 47.48 | 24.59 | 2.30 | -20.65 | 2.52 |
| AGACTTGGCATACACAC | RPS6KA3 | 24.93 | 19.63 | 1.08 | -23.15 | 2.48 |
| TTTTAATCTTGTTGGGA | KIAA1128 | 20.30 | 5.45 | 0.00 | -CIN III | 3.00 |
| AACCCTTGGCTGTAGTT | CRISP3 | 24.36 | 5.88 | 0.00 | -CIN III | 2.40 |
| TTTTTCAATCAAATTTG | MAP2 | 20.19 | 2.65 | 0.00 | -CIN III | 2.18 |
