# Supplementary material for: Up regulation in gene expression of chromatin remodelling factors in cervical intraepithelial neoplasia
Source: BMC Genomics. 2008 Feb 4;9:64. doi: 10.1186/1471-2164-9-64 (PMC2277413; doi:10.1186/1471-2164-9-64)
Supplement: Additional file 7 — Pathology on all cases. Pathology on all cases used as described by pathologist at the British Columbia Cancer Agency. [file 1471-2164-9-64-S7.doc]

Supplemental Table 6. Pathology evaluation of samples.

| **Library** | **Age** | **Description** |
| --- | --- | --- |
| N3 |  | Endocervix with squamous metaplasia and acute/chronic inflammation |
| N1 | 45 | LEEP cone biopsy uterine cervix: No residual dysplasia |
| N2 | 24 | negative for dysplasia, mature squamous epithelium, cervical stoma unremarkable |
| N4 | 55 | negative for dysplasia, unremarkable endocervical glands and stroma |
| M1 | 30 | mild squamous dysplasia (CIN I), extending to the exocervical resection margin in one block |
| M2 | 30 | Mild squamous dysplasia (CIN I) - fully excised |
| M3 | 31 | metaplastic and focally mildy dysplastic squamous epithelium (CIN I) - completely excised |
| M4 | 24 | near circumferential moderate squamous dysplasia (CIN II) - completely removed |
| M5 | 27 | LEEP biopsy contains moderate squamous dysplasia. No biopsy margin involvement |
| M6 | 21 | LEEP biopsy contains moderate squamous dysplasia. No biopsy margin involvement |
| C1 | 42 | severe dysplasia of squamous epithelium |
| C3 | 31 | CIN III, some ass w extension into cervical glands |
| C2 | 23 | severe dysplasia of squamous epithelium, exocervix |
| C4 | 28 | severe dyplasia (CIN III), CIN III does not extend to the endo or ectocervical margins. No invasion evident |
| C5 | 30 | severe dysplasia of the squamous epithelium extending focally to endocervical and exocervical resection margins |
| C6 | 23 | ectocervix with severe dysplasia (CIN III), benign endocervix - no invasion |
